# Wingless Directly Represses DPP Morphogen Expression via an Armadillo/TCF/Brinker Complex

**DOI:** 10.1371/journal.pone.0000142

**Published:** 2007-01-03

**Authors:** Heidi Theisen, Adeela Syed, Baochi T. Nguyen, Tamas Lukacsovich, Judith Purcell, Gyan Prakash Srivastava, David Iron, Karin Gaudenz, Qing Nie, Frederic Y.M. Wan, Marian L. Waterman, J. Lawrence Marsh

**Affiliations:** 1 Department of Developmental and Cell Biology, University of California Irvine, Irvine, California, United States of America; 2 Department of Mathematics, University of California Irvine, Irvine, California, United States of America; 3 Department of Microbiology and Molecular Genetics, University of California Irvine, Irvine, California, United States of America; 4 Developmental Biology Center, University of California Irvine, Irvine, California, United States of America; Max Planck Institute of Molecular Cell Biology and Genetics, Germany

## Abstract

**Background:**

Spatially restricted morphogen expression drives many patterning and regeneration processes, but how is the pattern of morphogen expression established and maintained? Patterning of *Drosophila* leg imaginal discs requires expression of the DPP morphogen dorsally and the wingless (WG) morphogen ventrally. We have shown that these mutually exclusive patterns of expression are controlled by a self-organizing system of feedback loops that involve WG and DPP, but whether the feedback is direct or indirect is not known.

**Methods/Findings:**

By analyzing expression patterns of regulatory DNA driving reporter genes in different genetic backgrounds, we identify a key component of this system by showing that WG directly represses transcription of the *dpp* gene in the ventral leg disc. Repression of *dpp* requires a tri-partite complex of the WG mediators armadillo (ARM) and dTCF, and the co-repressor Brinker, (BRK), wherein ARM•dTCF and BRK bind to independent sites within the *dpp* locus.

**Conclusions/Significance:**

Many examples of dTCF repression in the absence of WNT signaling have been described, but few examples of signal-driven repression requiring both ARM and dTCF binding have been reported. Thus, our findings represent a new mode of WG mediated repression and demonstrate that direct regulation between morphogen signaling pathways can contribute to a robust self-organizing system capable of dynamically maintaining territories of morphogen expression.

## Introduction

Numerous studies have demonstrated that WNT signaling (WG in Drosophila) mobilizes a nuclear β-catenin/TCF complex that can activate transcription of WNT target genes [1]–[4]. WNT signaling typically leads to the stabilization and nuclear accumulation of ß-catenin ARM (Armadillo), which forms an activating complex with the DNA binding WNT effector TCF (Pangolin or dTCF in Drosophila) [5]. However WNT signaling can also repress gene expression, even within the same cell where WNT activation occurs. In most cases it is unclear if repression is direct or indirect and the molecular mechanisms involved are unknown.

Development of the Drosophila leg imaginal disc requires maintaining complementary territories of dorsal *dpp* and ventral *wg* morphogen expression. We and others have noted that WNT/WG signaling activates *wg* expression and represses *dpp* expression in the ventral territory of the Drosophila leg imaginal disc, and this is critical for normal patterning of the disc [6]–[11], but whether WNT/WG directs ARM•dTCF complexes to activate expression of repressor proteins or whether ARM•dTCF complexes bind directly to the *dpp* gene to repress transcription is unclear. Here we investigate the mechanism of WG mediated repression of *dpp* and the basis of the self-organizing behavior of the *wg* and *dpp* expression territories (Theisen et al., 1996).

Studies with cultured cells using the WNT activated TOPFLASH promoter have identified many components that contribute to WNT mediated gene activation. However, the response to WG signaling *in vivo* is often repression of gene expression *e.g*. the *dpp*, *dfrizzled2* (*dfz2*), *stripe* (*sr*), *engrailed* (*en*), *ovo/shavenbaby* (*svb*), and Ubx genes are all repressed upon WG signaling [12]–[18]. It is not known if repression is direct or indirect and little is known about the co-effectors that produce an inhibitory signal versus an activating signal in response to WG signaling. To determine whether repression by WG signaling is direct or indirect and to better understand the factors that allow a WG signal to be inhibitory, we investigated whether dTCF binds to the *dpp* gene and whether dTCF and/or ARM are required for WG directed repression.

Here, we show that a novel WG dependent repressing complex that includes ARM•dTCF and the co-repressor Brinker binds directly to the *dpp* enhancer region to provide a key component of a self organizing regulatory loop.

## Results

### Identifying a WG response element in the *dpp* regulatory domain

**Figure 1:**
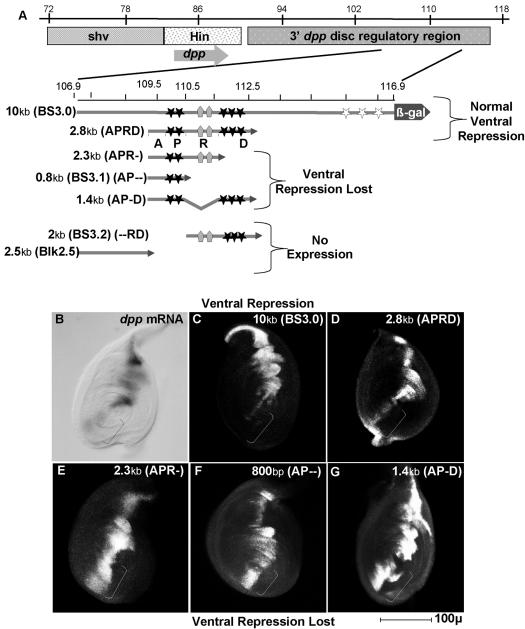
A 2.8 kb fragment of the dpp enhancer is sufficient for activation and repression of *dpp* in the leg disc. A: Schematic representation of the *dpp* locus and the 6 enhancer fragments used in this study. The *dpp* transcription unit is centered around 86 kb (arrow). [Map coordinates (in kilobases) from [19], [52], [53]. The leg disc enhancer is located between 20–30 kb downstream of the *dpp* coding region. Filled stars represent dTCF-binding sites confirmed by footprinting, open stars are predicted sites and pentagons are BRK binding sites. Arrowheads indicate fusion to the ß galactosidase reporter gene. APRD refers to the 4 relevant domains A (region required for Activation), P (proximal TCF sites), R (repressor domain), D (distal TCF sites). B–E: 3rd instar leg imaginal discs with dorsal up and anterior to the left. B: Normal *dpp* mRNA expression detected by *in situ* hybridization. Bracket indicates ventral region, where *dpp* is repressed. C: A 10 kb *dpp* enhancer fragment (BS3.0) drives expression of lacZ in a stripe that recapitulates normal *dpp* expression including ventral repression (bracket). D: Expression driven by the 2.8 kb APRD *dpp* enhancer fragment mimics *dpp* mRNA and BS3.0 expression. Again, note ventral repression (bracket). E: Ventral repression is lost (bracket) in the 2.3 kb APR- fragment which has a 500 bp region of APRD that contains the distal cluster of dTCF binding sites (D) deleted. F: An 800 bp fragment (AP--, BS3.1) containing the proximal cluster of dTCF sites (P) is not sufficient for ventral repression (bracket). G: The AP-D fragment does not show ventral repression (bracket). Sequences in the 1.4 kb between the proximal and distal dTCF sites do not contain dTCF sites but are required for ventral repression.

The *wg* and *dpp* genes are expressed in non-overlapping ventral and dorsal domains respectively in the leg imaginal disc of Drosophila. Loss of WG signaling leads to ectopic transcription of *dpp* and an engineered gain of WG signaling can suppress *dpp* transcription [6]–[11]. To determine if repression of *dpp* by WG is direct or indirect, we identified WG-responsive sequences within the *dpp* gene. The *dpp* gene is regulated by an extensive set of enhancers some of which are located approximately 30 kb downstream of the *dpp* coding region (Fig. 1A; [19]). A 10 kb fragment from this region (BS3.0; 106.9–116.9; Fig. 1A; [19]) directs β-galactosidase expression in the normal pattern of *dpp* expression in imaginal discs (Fig. 1B,C). In the leg disc, expression occurs in a stripe along the anterior/posterior (A/P) compartment boundary, except that extension of the stripe into the ventral region is prevented by WG-dependent repression (Fig. 1B,C) [6]–[11], [20], [21]. Since WG signaling is mediated via ARM•dTCF complexes, we scanned the 10 kb *dpp* enhancer fragment and found 8 potential dTCF binding sites [22], 5 of which fell into two clusters within 2kb of each other in a region that is able to direct expression in leg imaginal discs (Fig. 1A; APRD). A proximal cluster (P) is located around map coordinates 110 and is contained within fragments that activate *dpp* along the entire A/P boundary. Based on the location of these sites, we analyzed a series of *dpp* enhancer fragments in transgenic flies (Fig. 1A). At least 4 independent transformant lines were examined for each construct; and the expression patterns were the same for each line tested.

The smallest reporter construct that contains all the elements necessary to mimic the normal *dpp* expression pattern is a 2.8 kb *dpp* enhancer fragment that includes an activating region (A), the proximal dTCF cluster (P), a co-repressor binding region (R), and a distal cluster of dTCFsites (D) (APRD; 109.5–112.3) (Fig. 1D). We designate these four functional regions of the 2.8 kb enhancer as APRD with dashes to denote deletion of particular regions and lower case italics to denote regions in which specific dTCF binding sites have been mutated.

An 800 bp fragment containing both the activating region (A), and the proximal cluster of dTCF sites (P) [(BS3.1, AP--) [19]; 109.5–110.3] activates transcription along the A/P boundary but does not exhibit ventral repression (Fig. 1F). The downstream 2 kb region (--RD), containing the putative co-repressor binding element (R), and the distal cluster of dTCF sites (D), is required for repression but cannot itself activate expression [BS3.2 [19]; 110.3–112.3; Fig. 1A; data not shown]. Deleting the 1.4 kb R region of DNA between the dTCF clusters (AP-D)(Fig. 1A;G) or removing a 500 bp fragment that contains the distal cluster of dTCF sites (APR-)(Fig. 1A;E), results in loss of ventral repression. These data show that repression requires at least two regions in the adjacent 2 kb, namely the distal cluster of dTCF sites (D) and a co-repressing region (R) that does not contain dTCF sites. Genomic fragments that lack the 800 nucleotide AP fragment (Fig. 1A, --RD, BS3.2 of Blackman) are not expressed at all and hence repression cannot be evaluated [*e.g.* Blk2.5; 106.9–109.3, and BS3.2, [19] Fig. 1A; data not shown]. Thus, the minimal region necessary for proper *dpp* regulation in the leg disc is the 2.8 kb APRD fragment that contains distinct activating (A) and repressing sequences (RD).

### The 2.8 kb *dpp* enhancer, APRD, responds to WG signaling

**Figure 2:**
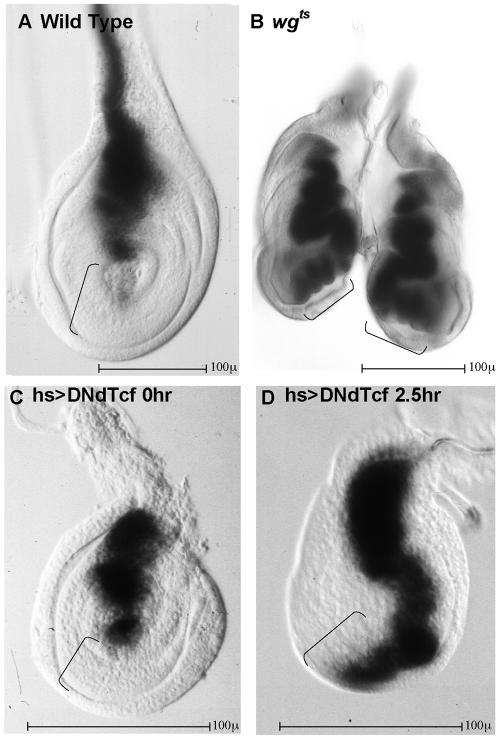
The *dpp* enhancer responds to WG signaling A–D: 3rd instar leg imaginal discs. Dorsal is up, anterior is to the left. Expression of the 2.8 kb APRD reporter fragment is monitored by β-galactosidase activity. A: In wild type leg discs (mesothoracic shown), APRD>LacZ expression is repressed in the ventral region (bracket). B: WG signaling is required for ventral repression. In a pair of everting prothoracic leg discs from a *wg^ts^* larva, ventral repression of APRD>LacZ is lost after shifting to restrictive temperature (brackets). C: Expression of the APRD reporter is repressed ventrally in Hs>Gal4; UAS>DNdTCF animals reared at 18° (bracket). DNdTCF is a dominant negative form of dTCF that cannot bind ARM. These animals and their discs are small compared to their non DNdTCF bearing sibs even when maintained continuously at low temperature, presumably due to low level expression of Hs>Gal4. However, these control animals maintained at low temperature do survive as viable, mophologically intact adults. D: When heat shocked in late third instar, repression is lost within 2.5 hours (bracket). At least 6 animals of each genotype were examined and all legs exhibited the same responses.

To determine if the *dpp* reporter constructs are responsive to WG signaling, we examined reporter gene expression in animals where WG signaling is blocked at the level of the ligand and at the level of ARM/dTCF. A temperature sensitive *wg* allele, *wg^IL114^* [23], was used to test the effect of WG signaling on the expression of both the 10 kb (BS3.0) and the 2.8 kb *dpp* enhancer (APRD) fragments (Fig. 2A,B). Repression of both the 10 kb and 2.8 kb (APRD) *dpp* reporters is lost in the ventral region of *wg^ts^* discs within 24 h of a temperature shift, indicating that the APRD region of the *dpp* enhancer is responsive to WG directed repression (Fig. 2A,B and data not shown).

To block the nuclear response to WG signaling, we expressed dominant negative dTCF (DNdTCF), which lacks the ARM binding domain [22], and therefore acts as a nuclear repressor of the WG pathway. If repression of *dpp* by WG requires an ARM•dTCF complex, then over-expression of DNdTCF should block repression of *dpp* transcription and result in *dpp* expression in the ventral region. Expression of UAS>DNdTCF was driven with the HS>Gal4 driver and expression of the BS3.0 and APRD enhancer fragments was monitored. Within 2.5 hrs of activating DNdTCF by shifting to 25°C, expression of the *dpp* reporter increased dramatically in the ventral region (compare Fig. 2D vs C). The cell cycle time at this stage was ∼6–10 hrs [24], [25], therefore, the change in gene expression occurred over the course of ≤1 cell division, suggesting that the regulation of *dpp* gene expression by ARM•dTCF is not an indirect consequence of a regenerative response. To confirm that the endogenous *dpp* gene also responds to DNdTCF, *dpp* expression was monitored in animals where the *dpp^blink^*>Gal4 driver was used to drive DNdTCF expression in a pattern that overlaps both the dorsal region of *dpp* expression and the ventral region of *wg* expression in leg discs [26]. Repression of endogenous *dpp* is lost in these discs (not shown). Thus, blocking WG signaling either at the level of ligand activity or at the level of ARM•dTCF complex formation, leads to a rapid loss of *dpp* repression in ventral cells of the leg discs, indicating that repression of *dpp* transcription requires the formation of ARM•dTCF complexes.

### Repression of the *dpp* enhancer requires dTCF binding

**Figure 3:**
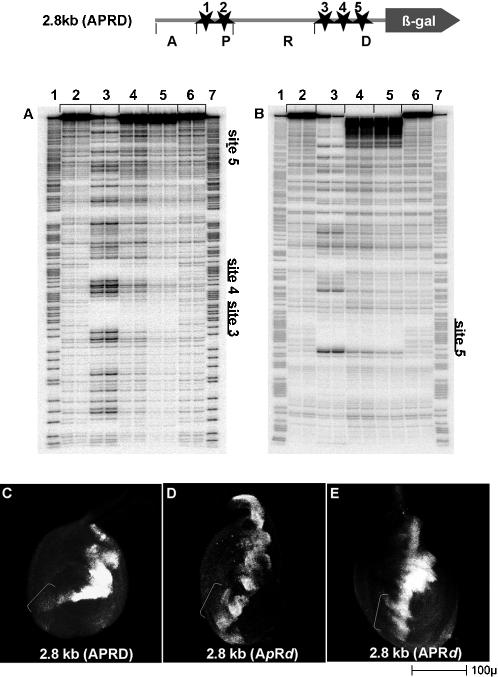
Identification of dTCF binding sites required for *dpp* ventral repression A,B: dTCF binding sites in the *dpp* regulatory region from 109.4–112.8 kb were mapped by DNase I footprinting using dTCF protein as described in the methods section [22]. The approximate positions of the protected sites are indicated by stars. DNase I footprinting of the region containing the distal cluster (D) reveals 3 protected sites (sites 3, 4; 5) indicated by the bars in A and B. Similar footprints identified two sites in the proximal cluster (sites 1; 2 = P) and no footprints or gel shifts were detected in the A or R regions (not shown). Duplicate lanes represent independent reactions. Lanes 1; 7 are the GA sequencing ladder. All lanes utilize a 1∶1 dilution of bacterial extract containing empty expression vector or protein expressing vector and the same concentration of DNaseI except lane 4. Lanes 2 and 6 are no protein controls. Lane 3 uses an extract expressing human LEF1 protein. Lanes 4 and 5 use an extract expressing dTCF with lane 4 containing a 3 times higher concentration of DNase. C–E: 3rd instar leg imaginal discs. Dorsal is up, anterior is to the left. *dpp* lacZ expression is monitored by immunofluorescence. C: The 2.8 kb APRD *dpp* enhancer fragment with all 5 dTCF sites intact is repressed ventrally (bracket). D: Mutation of all 5 dTCF sites (A*p*R*d*) eliminates ventral repression (bracket). E: Mutation of just the 3 distal dTCF sites (APR*d*) is sufficient to eliminate ventral repression (bracket).

To evaluate whether the rapid de-repression in response to DNdTCF reflects competition for dTCF binding sites within the *dpp* locus or an indirect effect being mediated through other factors, we sought to map and mutate the putative dTCF binding sites in the *dpp* regulatory region. DNAse I footprinting analysis with both recombinant dTCF protein and with human LEF-1 protein showed that both the Drosophila and human proteins protect all 5 putative TCF binding sites in the APRD *dpp* fragment (Fig. 3A, B and data not shown). We also performed electrophoretic mobility shift assays to confirm that these sites were the only *bona fide* dTCF binding sites and that there were no other dTCF binding sites within the APRD region (data not shown).

**Figure 4:**
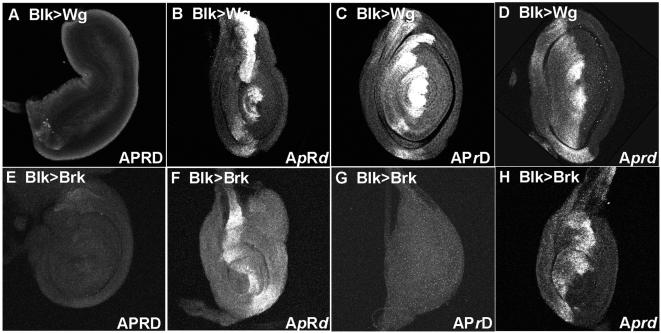
Simultaneous binding of BRK and dTCF is required for *dpp* repression. A–H: 3^rd^ instar leg imaginal discs. Dorsal is up and Anterior is to the left. A–D: response of *dpp* reporters to dpp^blk^ GAL4 driven expression of WG. E–H: response of *dpp* reporters to dpp^blk^ GAL4 driven expression of BRK. A: Ectopic dorsal expression of *wg* represses APRD>lacZ expression. B: Ectopic *wg* expression does not repress the APRD *dpp* reporter when all 5 of the dTCF binding sites are mutated (indicated by A*p*R*d*). C: WG expression does not repress the APRD *dpp* reporter when the BRK binding sites are mutated (AP*r*D). D: WG expression does not repress the APRD *dpp* reporter when all the dTCF and BRK binding sites are mutated (A*prd*). E: Ectopic dorsal expression of BRK represses APRD>lacZ expression. F: Ectopic BRK expression does not repress the APRD *dpp* reporter when all 5 of the dTCF binding sites are mutated (A*p*R*d*). G: Ectopic BRK expression does repress the *dpp* reporter when the BRK sites are mutated, APrD H: Ectopic BRK expression does not repress the *dpp* reporter when all the dTCF and BRK binding sites are mutated, A*prd*.

To test whether direct binding of dTCF to the 2.8 kb *dpp* enhancer fragment is required for *dpp* regulation, we engineered specific inactivating mutations in all 5 dTCF binding sites (A*p*R*d*) or only in the distal cluster of 3 dTCF sites (APR*d*). Gel shift experiments with recombinant dTCF demonstrated that the introduced mutations eliminated dTCF binding (data not shown). We compared the expression of the *dpp* reporter gene with the dTCF sites intact *vs.* mutated. Loss of binding sites either in both clusters or in only the distal cluster (A*p*R*d* or APR*d*), caused a dramatic loss of repression in the ventral leg disc (Fig. 3C–E). As described earlier, the two dTCF sites in the Proximal Cluster of the APRD fragment are not sufficient to cause measurable repression when the distal complex is absent nor are TCF sites required for activation since fragments with all TCF sites mutated still drive expression (not shown). These data demonstrate that binding of dTCF to the distal sites is necessary to inhibit *dpp* transcription. This is further confirmed by finding that mutation of the dTCF sites leads to ventral expression that is unresponsive to WG, ARM and dTCF overexpression (Fig. 4A, B and data not shown). Thus, functional dTCF binding sites in the APRD *dpp* enhancer fragment are required for WG dependent repression of *dpp* transcription *in vivo*.

### Brinker is required for WG dependent repression of *dpp*

How is it that dTCF binding in response to WG signaling inhibits expression of *dpp* but activates other genes? The AP-D construct, which contains 5 intact dTCF sites but has an internal deletion (Fig. 1G), has lost repression in the ventral region of the leg disc. This suggests that the deleted region contains an element that cooperates with dTCF to repress *dpp* transcription. A scan of this co-repressor region (R) for potential binding sites of known repressors of *dpp* identified two potential Brinker (BRK) sites. BRK is a sequence-specific transcription factor that is repressed by DPP signaling. Furthermore, the expression pattern of *brk* compliments that of *dpp* in the leg disk; there is lower expression along the A/P boundary in the dorsal region, but strong expression in the anterior and posterior regions, and intermediate expression along the A/P boundary in the ventral leg disk [27]–[30].

**Figure 5:**
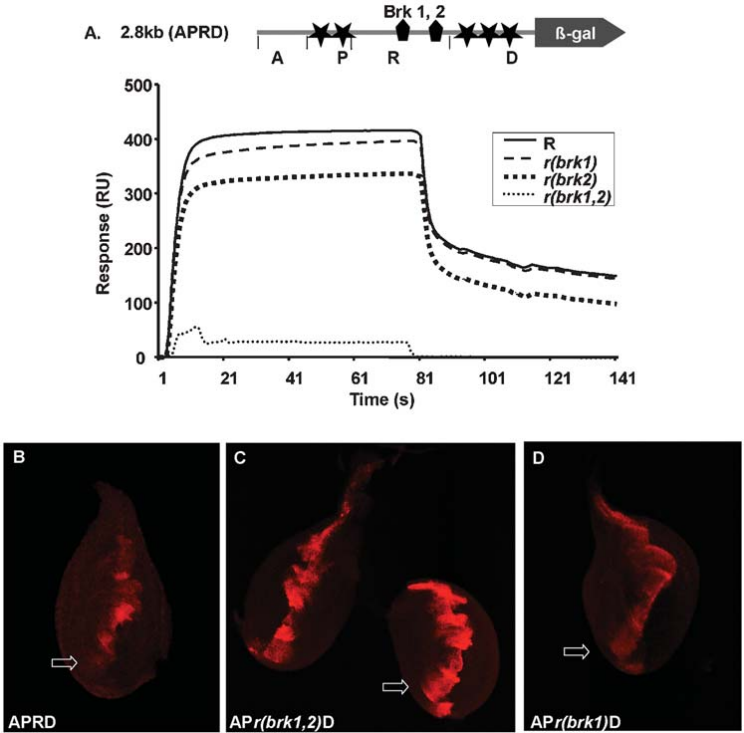
BRK binding is required to suppress *dpp* expression BRK binding sites are located in the R domain of APRD (filled pentagons). SPR analysis shows BRK binding to the intact R domain (R). Mutation of BRK site 1 [*r(brk1)*] reduces binding incrementally, mutation of BRK site 2 [*r(brk2)*] reduces binding still further while mutation of both sites [*r(brk1,2)*] abolishes binding completely. The biophysical binding of BRK to its DNA sites correlates well with the biological responses caused by the same mutations. B: *dpp* expression is ventrally repressed in the intact APRD fragment (arrow). C: Mutation of both BRK sites leads to loss of repression and ventral expression of *dpp* (arrow). D: Mutation of a single BRK site leads to ventral expression of *dpp* (arrow).

To test whether BRK binds to both of the potential sites in the R region, we used surface plasmon resonance (SPR) with immobilized recombinant BRK protein in a DNA binding assay (Fig. 5A). The SPR sensogram shows that BRK can bind to the R region when at least one of the BRK binding sites is intact, but when both BRK sites are mutated, no binding is observed.

If BRK is specifically required for WG mediated repression of *dpp*, then introducing either or both mutations into the BRK sites (AP*rb1*D, AP*rb2*D, and AP*rb12*D) should lead to increased *dpp* expression in the ventral region of the leg disk. Indeed, mutation of either BRK site 1 or both sites, results in increased *dpp* expression that is restricted to the region of WG signaling (Fig. 5 B,C,D).

To determine whether BRK binding is an essential component of WG mediated *dpp* repression, we tested the ability of WG signaling to repress reporter constructs when the BRK sites are mutated. While ectopic *wg* expression is able to extinguish all APRD expression (Fig. 4A), ectopic WG cannot repress APRD when the BRK sites are mutated (AP*r*D) (Fig. 4C). Nor can ectopic WG suppress reporter gene expression when the dTCF sites are mutated (A*p*R*d*; Fig. 4B) or when both the dTCF and BRK sites are mutated (A*prd*) (Fig. 4D).

To investigate the interdependence of WG and BRK, we asked if BRK alone is sufficient to repress expression of the *dpp* reporter. Ectopic *brk* expression can repress intact APRD (Fig. 4E), but cannot repress APRD when the TCF sites are mutated (A*p*R*d*; Fig. 4F) indicating that BRK must synergize with TCF to repress *dpp* expression. Interestingly, high levels of ectopic BRK can repress APRD even when the BRK sites are mutated (AP*r*D; Fig. 4G) but only if the dTCF sites are intact (A*prd*; Fig. 4H; F). This suggests that under normal cellular conditions, loss of BRK binding sites prevents repressor complex formation but that experimental induction of high levels of BRK may allow repressor complexes to form that are anchored to the DNA by dTCF•ARM complexes. Taken together these data suggest that at normal factor concentrations both BRK and dTCF sites are necessary for WG mediated repression of *dpp* transcription but neither alone is sufficient.

## Discussion

### Active Repression of *dpp* by WG defines a novel mode of WG mediated repression

TCF is emerging as a multifunctional transcriptional modulator that can act as both an activator and a repressor in multiple environments. In the absence of WNT signaling, LEF/TCFs become default repressors [4], [31]–[33] of genes because they recruit co-repressors such as GRO and CtBP [13], [34]–[36]. WNT signaling relieves this repression by causing β-catenin/ARM to accumulate in the nucleus and convert dTCF to a transcriptional activator, possibly by displacing or overriding the default co-repressor(s) [37]. This default repression can be further modulated by processes that antagonize the interaction of β-catenin with TCF.

Less well understood is the mechanism whereby TCF can repress genes in response to Wnt signaling. Expression of several genes is repressed in response to WNT signaling, including, *E-cadherin, dpp, Ubx, osteocalcin, stripe, svb, daughterless* [14]–[17], [38]–[43]. Thus far, one mechanism for WG/WNT dependent repression has been described namely, Competitive Repression [44]. In this case, dTCF represses gene activation by displacing other activating proteins through competition for the DNA binding site. For example, WG signaling represses *stripe* gene expression when dTCF binds to sites that overlap with the sites for the activator (CI) [15]. TCF has also been shown to mask the DNA binding domain of another transcription activator Runt and inhibit its binding to the osteocalcin promoter [42]. In both these cases, repression occurs in response to the WG/WNT signal and requires ARM. Here, we provide evidence of a second mechanism of WG/WNT directed repression, namely Direct Repression [44]. We show, for the first time, that WNT signaling can direct formation of a co-R•ARM•TCF complex that represses transcription. In the case of *dpp* repression, this co-R is BRK and the formation of a BRK•ARM•dTCF complex is required to actively repress *dpp* gene expression. Other genes, including *ovo/svb, da* and *dfz2* in Drosophila, are actively repressed by WG signaling and contain physically separated activating and repressing enhancer elements [12], [14], [38], but since the putative regulatory DNA regions necessary for repression of these genes have not been identified, it is not yet possible to tell if repression in these cases also requires an ARM•TCF complex.

Our studies show that BRK can interact with the dTCF•ARM complex to repress target genes. The behavior of the complex in response to altered levels of individual components, especially to altered levels of the non-DNA binding component, ARM, is not monotonic (e.g. repression is lost with both low and artificially high levels of ARM), suggesting a mechanism whereby both TCF and BRK can be titrated out by excess ARM which might be achieved by either direct or indirect interaction of ARM with both DNA binding components. Although, the specific molecular interactions that dictate the behavior of this complex remain to be determined, one can imagine several scenarios. To better understand the potential implications of these different scenarios, we constructed mathematical models that differ primarily in the nature of the interactions between DNA binding and non-binding components (Fig. S1–[Supplementary-material pone.0000142.s004] [Supplementary-material pone.0000142.s005] [Supplementary-material pone.0000142.s006] S5 ). This modeling analysis suggests distinct functional responses to different biochemical mechanisms that will be the subject of future studies. The biological responses described here and our analysis by modeling using reported values for the biophysical parameters [54]–[61], (Supporting Text S1; Figs. S1–[Supplementary-material pone.0000142.s004] [Supplementary-material pone.0000142.s005] [Supplementary-material pone.0000142.s006] [Supplementary-material pone.0000142.s007] S6 and Table S1 ) suggest a possible interaction mechanism in which a single ARM protein interacts either directly or indirectly with both TCF and BRK.

Since the *brk* gene appears to have no mammalian homolog, a different co-R could convert dTCF•ARM to a repressor complex in mammalian systems. The properties of this tri-partite repressor system are unique compared to the other known mechanisms of WG repression in that rather than being monotonic with respect to changes in all components, the system exhibits an optimum with respect to ARM levels. Systems with such properties tend to self-correct. For example, as ARM increases, *dpp* repression increases until ARM levels reach a point where they start to form non-productive complexes (e.g. increasing ARM positively feeds back on WG expression which coupled with less *dpp* allows greater levels of WG signaling and stabilized ARM). Higher levels of ARM will lead to the formation of non-productive complexes and squelching (Figs. S1Ci and S2; S5 ) and *dpp* repression will decline. Subsequent elevation of *dpp* expression will negatively affect WG signaling and ARM levels will correct back toward their optimum.

During development, it is essential for organ anlage such as imaginal discs in Drosophila or limb blastema in vertebrates, to develop the asymmetry required to produce a chiral appendage such as a leg. In imaginal discs, compartments of lineage restriction provide one axis of asymmetry along the A/P axis but no evidence for lineage restricted regions has been found in other axes such as the D/V axis of legs or antennae. How then are the dorsal and ventral territories defined and maintained? The system of mutual repression between Wg and Dpp described here, provides a mechanism for maintaining separate "territories" of *wg* and *dpp* expression in a developing field. Territories are regions of cells that are under the domineering influence of a particular morphogen and they differ from compartments in that they are not defined by lineage but are dynamically maintained by continuous morphogen signaling [11].

When targeted to an opposing morphogen gene (e.g. *dpp*), the properties of this novel BRK based co-repressor system contribute to a robust self organizing system that is capable of ensuring that territories of *wg* and *dpp* expression remain distinct and are maintained intact during the processes of growth and regeneration [10]; thus providing a molecular basis for the maintenance of such dynamic territories. Cross inhibition of morphogen expression may play a role in several developing systems including mammalian systems as similar repression of BMP by WNTs has been observed in the mammalian hair follicle and crypts of the developing gut [45].

## Materials and Methods

### *Drosophila melanogaster* stocks and crosses

Genetic markers are described in Lindsay and Zimm [46]. Ectopic expression experiments employed the *dpp^blk^*>Gal4 driver, P[GAL4-dpp.blk1 w+mW.hs]39B2/*TM6B* [26], and the HS>GAL4, P[GAL4-Hsp70.PB] driver mated to the following transgenes P[UAS>ARM52] (a kind gift of M. Peifer), P[UAS>dTCF] and P[UAS>DNdTCF] [22]. To enhance larval survival, animals were raised at low temperature until late 2^nd^/early 3^rd^ instar and then shifted to 29°C. The *dpp^blink^*>Gal4; UAS>dTCF animals were raised at 22°C and upshifted to 29°C for 3 h, 6 h, 12 h, 24 h and 48 h before dissection and staining of late 3^rd^ instar imaginal discs. Similarly, *dpp^blink^*>Gal4; UAS>DNdTCF animals were raised at 18°C and shifted to 25°C for 3 h, 6 h, 12 h and 24 h before dissection and staining. The crosses included various *dpp-lac*Z reporters as indicated in the text. For the *dpp^blk^*>Gal4 crosses, balancers with *Green Flourescent Protein* (*GFP*) were used to identify larvae for dissection. The *dpp*>*lac*Z reporter lines used were BS3.0, BS3.1 (AP--), BS3.2 (--RD)(kind gifts from Ron Blackman; [19] as well as APRD, APR- and AP-D (Fig. 1A). The APRD construct is a 2.8 kb *Hind*III-*Nhe*I fragment that starts 2.6 kb 3′ from the beginning of BS3.0 (*i.e.* at co-ordinate 109.5). APR- is a 2.3 kb *Hin*d III-*Bsa* B1 fragment that has the same start point as APRD. The AP-D construct was generated by ligating a 525 bp *Ssp*I-*Nhe*I fragment containing three dTCF binding sites (co-ordinates 111.8–112.5) to the 5′ end of APRD cut with *Hind*III-*Ssp*I (Fig. 1A). APRD and BS3.0 expression were also monitored in a temperature sensitive *wg* background. The temperature sensitive *wg* allele, *wg^IL114^* [23] was balanced with the compound balancer chromosome TSTL that has a translocation between the CyO and TM6B, *Tb* balancers. Homozygous mutant larvae were identified by the absence of a *Tubby* phenotype. The *wg^ts^* mutant animals were raised at 18°C and shifted to 25°C for 24–48 hrs before dissection in late third instar.

### Histochemistry

Imaginal discs were stained for β-galactosidase activity and mounted as described [7] with 2 minutes fixation. Expression was monitored in legs from at least 6 animals. The same changes in gene expression were observed in all animals with a particular genotype.

### In situ hybridizations

*wg* and *dpp* expression were monitored by whole mount *in situ* hybridization using digoxigenin labeled antisense RNA probes prepared according to the manufacturer's specifications (Roche Molecular Biochemicals). Plasmids used were a 3 kb *wg* cDNA (wg651, a kind gift of B. Cohen) and a 4 kb *dpp* cDNA dppE55 [47] both in bluescript. Prehybridization and hybridization conditions are based on the protocol of Tautz and Pfeifle [48] with modifications [11].

### Immunohistochemistry

Imaginal discs were fixed as for *in situs* and incubated overnight at 4°C with rabbit anti β-galactosidase antibody diluted 1∶1000 with PBT (PBS+0.1% Triton×100)+3%BSA. A Cy3 or FITC conjugated donkey secondary antibody (Jackson Immunological Laboratory) was used at a 1/200 dilution. Images were analyzed on a Zeiss 510Meta confocal microscope. In each experiment, gene expression was monitored in legs from at least 6 animals each from 4 transgenic lines. The same changes in gene expression were observed in all animals with a particular genotype.

### Protein Preparation and DNAse I footprinting

The DNA binding domain of dTCF was amplified by PCR using primers 5′CGCGGATCCGGAAGCAAAGCACACATCA, and 5′CGCGGATCCGCACCACTG ACTCTGTTG, and cloned into pET15b (Novagen). Bacterial extracts were prepared as described in [49]. Recombinant hLEF-1 [50] and dTCF were incubated with double-stranded DNA probes (5 to 15 fmol per reaction; single end-labeled on the 5′ end with [γ-^32^P] ATP) for 1 minute on ice in a 50 µl reaction containing TM buffer (50 mM Tris pH 7.9, 12.5 mM MgCl_2_, 1 mM EDTA, 20% glycerol, 0.1% NP-40, 50 mM KCl). DNase I work-up procedures are described in [51]. Human LEF-1 footprinted to the same sites as dTCF as expected from the highly similar DNA binding domains of these proteins [22]. All gels were analyzed with a PhosphorImager (Molecular Dynamics).

### Mutation of dTCF and Brk binding sites

Site-directed mutants were made using the *Pfu* mutagenesis kit (Stratagene) with two complementary 30 nucleotide primers containing the new sequence. Approximately two-thirds of the colonies picked were the correct mutant. The sites were mutated as listed, wild type sequence is underlined, and mutated sequence is in capitals: (site 1) aacttctttcaa>aacttcttCGaa; (site 2) aacttctttcag>aacttcttCcag; (site 3) catcaatggcag>catTCatggcag; (site 4) gtacaaagaccc>gtaTGaagaccc; (site 5) tgccttttgatg>tgcctttATatg.

To mutate the BRK binding sites the following mutagenic oligonucleotides were used (the BRK site or its complement is shown with bold letters with the altered nucleotides underlined):
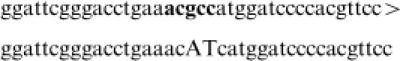
and
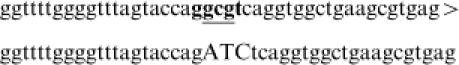
The first mutation eliminates an *Nco*I site (ccatgg) while the second mutation creates a *Bgl*II site (agatct) making the detection of the mutations easier.

### Surface Plasmon Resonance

Computational scanning of 2.8 kb APRD region revealed two consensus BRK binding sites. These were functionally confirmed by SPR on a Biacore 3000. Carboxymethylated dextran (CM5) coated sensor chips (Biacore AB, Uppsala, Sweden) were coated with 800 response units of anti-Flag antibody (Sigma) using NHS/EDC chemistry. HBS buffer (10 mM HEPES pH 7.4, 0.15 M NaCl, 3 mM EDTA, 0.005% (v/v) Surfactant P20; Biacore AB) was used as the running buffer with a flow rate of 10 µl/min. A fusion protein of the BRK-DNA binding domain with a FLAG epitope tag was purified [16] and captured onto the anti-Flag antibody. A 560 bp fragment spanning both putative BRK sites was tested for binding to immobilized BRK protein and binding was demonstrated. The role of the specific BRK sites was confirmed by mutating each site alone and both together within the context of the 560 bp fragment. Mutation of either BRK site reduced (slightly) but did not eliminate binding while mutation of both sites resulted in no detectable binding. The surface was regenerated with 2×5 µl of 30 mM HCl. The sensorgram for soluble antigen binding was corrected with the control buffer sensorgram

## Supporting Information

Supporting Text S1 (0.82 MB DOC)

Table S1 Descriptions, values, and references of parameters used. (6.76 MB PDF)

Figure S1 Computational analysis activation/repression responses of wg and dpp under different possible modes of action A: Cartoon key for the 3 proteins and DNA binding sites involved. The wg enhancer (e3) serves to activate wg expression, while the dpp enhancer (e1e2) contains both TCF (e1) and BRK (e2) binding sites and is repressed by WG signaling. Both TCF and BRK bind DNA while ARM does not. B: (i) Depicts the TCF based activation complex formed at the wg enhancer (ii) depicts 3 possible models of complexes involving TCF, BRK and ARM that might contribute to repression. Model 1 requires concurrent binding of an ARM•dTCF complex and BRK but no physical interaction. Model 2 postulates that repression of dpp requires a bridge between TCF and BRK that requires ARM (bridging model). Model 3 proposes a direct binding between TCF and BRK. C(i) Examples of non-productive complexes that might form in the presence of high levels of A under the bridging model (1) or that might form in the presence of high levels of T in the direct binding model (2) (ii) examples of the possible sequences of binding events under model 1. There are several possible intermediates on the way to productive complexes (ATe3 or e1TABe2). D: The system is experimentally manipulated by increasing or decreasing the production rates (VT, VA, or VB) of T, A, or B. The computationally predicted response of wg activation (dashed line) and dpp repression (solid line) to changing levels of T, A or B expression is plotted over a wide range of production rates. The experimentally observed response of wild type dpp (e) and wg (f) expression to increased levels of ARM production (g, h) and TCF production (i, j) is shown in the bottom panels. The qualitative behavior predicted by the computational analysis disagrees with the concurrent binding and direct T•A binding models but is consistent with the bridging model when non-productive complexes are considered.
(6.41 MB PDF)

Figure S2 All possible protein-protein and protein-DNA interactions for activation of wg and repression of dpp by models (1) and (2) are shown. Cartoons illustrate the interactions in question and the corresponding binding equations are listed to the right. A. Reactions leading to activation of wg are shown. B. Binding reactions for the concurrent binding model (model 1) are shown where the T•A complex does not bind B. C. Additional binding reactions describing events corresponding to the bridging model (model 2) are shown in a dashed box that correlates with equations in Fig. S3. These binding reactions together with those in B comprise the full set of reactions for the bridging model (2) without formation of NPCs. D. The binding reactions shown in the solid-box describe the formation of all possible NPCs. Together with the reactions shown in B and C, they comprise the full set of reactions for the bridging model with non-productive complexes. Transcriptionally active complexes are shown in bold.
(6.24 MB PDF)

Figure S3 The equations governing activation and repression models (1) and (2) are shown. The unboxed, dash-boxed, and solid-boxed equations/terms correspond to the unboxed, dash-boxed, and solid-boxed interactions in Fig. S2. Model 1 (concurrent binding) is described by the set of equations not enclosed in the dashed and solid-boxes. Model 2 (ARM bridging) is described by the full set of equations. Omitting the terms in the solid-box describes the bridging model (2) in the absence of the formation of NPCs.
(6.24 MB PDF)

Figure S4 All possible protein-protein and protein-DNA interactions for activation of wg and repression of dpp by the direct binding model (models 3) are shown. Several binding reactions in this model are possible intermediates enroute to final complexes and are identical to binding events shown for other models above. A. Describes the wg activation reactions as in Fig. S2). B. Describes intermediate reactions that are the same as the concurrent binding reactions. C. Binding reactions unique to the T•B binding model are shown in the dashed box. D. The binding reactions leading to non-productive complexes in the T•B binding scenario are shown in the solid box. Transcriptionally active complexes are shown in bold.
(6.24 MB PDF)

Figure S5 Equations governing repression by direct T•B binding (model 3) are shown. The complete set of equations describes the behavior of the direct T•B binding reactions in Fig. S4 with the inclusion of non-productive complexes. Omitting the terms in the solid-box describes the behavior under this model (3) in the absence of the formation of NPCs.
(6.24 MB PDF)

Figure S6 Comparison of the response of T and B to increasing production rates. Why is the response to increased production rate of T to squelch T mediated regulation while increasing production rate of B has little effect? The lack of a known feedback on production of T leads to rapid change in the T:A ratio while the known feedback loops governing levels of B tend to maintain a steady ratio of B:A.
(6.24 MB PDF)
