# Supplementary material for: Wingless Directly Represses DPP Morphogen Expression via an Armadillo/TCF/Brinker Complex
Source: PLoS One. 2007 Jan 3;2(1):e142. doi: 10.1371/journal.pone.0000142 (PMC1764032; doi:10.1371/journal.pone.0000142)
Supplement: Supporting Text S1 — (0.82 MB DOC) [file pone.0000142.s001.doc]

**Supporting Information for**

# **Wingless Directly Represses DPP Morphogen Expression Via an Armadillo/TCF/Brinker Complex**

Theisen et. al.

**Theoretical exploration of the molecular interactions governing repression**

Although many signaling pathways revert to a default repression state in the absence of signal [1], cooperative interactions that convert an activating WG signal into a repressing one have not been reported. Our experimental observations show that the response of the *dpp* system to changes in ARM levels is not monotonic; *dpp* repression is lost at both low and high levels of ARM. This raises the possibility of a novel interaction between the ARM•TCF complex and BRK.

To better understand how the biochemical interactions of these components might affect biological behavior, we decided to explore different scenarios for such a system *in silico*. We developed a series of ordinary differental equations (ODEs) to describe the possible interactions between two DNA binding proteins, a non-DNA binding protein and their DNA enhancer target sites *e_1,2,3_*. Three mechanistic possibilities for their interaction were considered (Fig. S1B) and predicted behavior was compared with experimentally observed behavior. This analysis compared the behavior of an enhancer that positively responds to WG signaling such as the dTCF binding region of the *wg* enhancer that leads to *wg* activation (represented by *e_3_*), with an enhancer (*e_1_e_2_*) that negatively responds to WG signaling such as the *dpp* repression region with dTCF binding sites (*e_1_*) and BRK binding sites (*e_2_*) (Fig. S1A). Binding of AT to *e_3_* activates *wg* (Fig. S1Bi), while repression of *dpp* requires A, T and B bound to *e_1_e_2_* (Fig. S1Bii). To explore the dynamics of these possible systems, all possible molecular interactions were included without bias. Equilibria and rate constants derived from known measured values and known feedback loops are documented below.

We considered three possible models for the interaction of ARM, TCF and BRK in repressing *dpp* expression (Fig. S1): (1) In case 1, the concurrent binding model, an ARM•TCF complex and BRK bind DNA independently to cause repression but there is no physical interaction between them (Fig. S1Bii model1). (2) In case 2, the bridging model, ARM participates either directly or indirectly in forming a bridge that binds both BRK and TCF to form a repressing complex *e_1_*TAB*e_2_* (Fig. S1Bii model2). In this scenario, ARM acts like a scaffold with at least 2 binding surfaces, one for TCF and one for BRK or for an intermediate that binds BRK. (3) In case 3, the direct T•B binding model, direct binding between BRK and dTCF occurs without the participation of a non-DNA binding element (Fig. S1Bii model 3). These putative interactions do not exclude the possibility of additional elements, but the major distinction between the three scenarios is whether independent DNA binding elements interact directly or whether a non-DNA binding bridge is required.

The response of *wg* and *dpp* to varying levels of ARM, TCF and BRK, was analyzed in the context of each of the 3 mechanistic models. One modeling scenario allowed all possible mass action binding events, which permits the formation of non-productive complexes (NPCs; Fig. S1Ci), while a second scenario eliminated the formation of non-productive complexes (NPCs). The modeling predicted different experimental responses for each of the different mechanisms (Fig. S1D), with the most prominent distinction being the response of activation and repression to excess levels of ARM and dTCF.

This analysis demonstrates that different formalized mechanisms of repression can be distinguished by the response of the system to changes in the levels of components and in this case changes in the levels of non-DNA binding components (e.g. ARM) provide the distinguishing behaviors. Experimentally we observe that over-expression of ARM (using Blk>Gal4 to drive UAS>ARM in a D/V stripe in the leg imaginal disc) causes both the APRD *dpp* reporter gene and endogenous *dpp* mRNA (Fig. S1G; data not shown) expression to expand into the ventral region of the leg disc. Endogenous *wg* mRNA expression (Fig. S1H) remains on in the ventral region but expands into the dorsal territory in these animals. Thus, over-expression of ARM activates WG target genes (e.g. *wg*) but squelches WG dependent repression. This squelching behavior [2] can help distinguish between biochemical mechanisms that depend only on the DNA binding components versus mechanisms in which a non-DNA binding component forms a bridge between the two DNA binding species [3,4] (Fig. S1Bii2). For example, direct binding between TCF and BRK predicts increased repression with increasing ARM (Fig. S1Dd), while models that do not include ARM•BRK or TCF•BRK interaction predict no change in repression upon increased ARM. Responses of the system to experimental changes in TCF and BRK levels also support a bridging model. For example, models that fail to include ARM•BRK binding or models that do not permit the formation of non-productive complexes, fail to anticipate the loss of repression seen with excess TCF. The reduced sensitivity of *dpp* repression to changes in BRK levels compared to changes in TCF levels also supports a bridging model. Both the experimentally observed changes in gene expression and the computational analysis suggest a possible bridging mechanism (Fig. S1Biib) for ARM, TCF and BRK mediated repression.

Materials and Methods

A series of ODEs describe the possible binding of A to T, A to B, B to T and T and B to their respective enhancers on the DNA (*e_1, 2_* _or_ *_3_*) (the complete set of ODEs is shown in Figs. S2-5) and these were used to explore different possible mechanisms of action of this system. We adopted the following abbreviations simplicity in the computational analysis: WG = W; DPP = D; ARM = A; dTCF = T; and BRK = B. dTCF binding sites in the *dpp* gene are represented by *e_1_* and BRK enhancer binding sites by *e_2_* and dTCF binding sites in the *wg* gene are represented by *e_3_* (Fig. S1A). These expressions incorporate variables for synthesis (*V_T, A_* _or_ *_B_*) and degradation of the components (K_deg_).

The concentrations of A and B are governed by feedback loops. To mimic the stabilization of ARM in response to WG signaling, the regulation of A which is governed by the degradation of ARM, *K_degA_,* is described by an equation in which A is constantly degraded but stabilized by WG signaling (i.e. increasing AT*e_3_*).

The Hill coefficient (m) imparts cooperativity to the interaction and gamma is a term that reflects the sensitivity of the system to feedback regulation.

To mimic repression of *brk* by DPP [5-7], decreasing D (increasing *e_1_*TAB*e_2_*), has a positive feedback on the production of B (*V_B_*), represented by,

where *V_B_* is the production rate of BRK, γ is the signaling sensitivity or EC50, the effective concentration so that maximal feedback occurs at the halfway point between the maximum and minimum response values, *m* and *n* are the Hill coefficients that provide a measure of the extent of cooperativity in binding, *K_Amax_* and *K_Amin_* are maximum and minimum degradation rates of ARM, and *V_Bmax_* and *V_Bmin_* are maximum and minimum production rates of BRK. ARM has a constant production rate denoted by$V_{A}$. To preserve the conservation of T as experimentally observed in the ventral leg disc cells, T is governed by a production rate (*V_T_*) and a degradation rate (K_degT_ = V_T_/T). To explore parameters, all molecules are allowed to interact randomly in any order with no bias.

Using these molecules, we modeled WG directed *dpp* repression and *wg* activation. To model repression, we consider three possible modes of ARM, TCF and BRK interactions (Fig. S1Bii): (1) An ARM•TCF complex binds to *e_1_* and BRK binds *e_2_* concurrently to cause repression (AT*e_1_e_2_*B) but there is no physical interaction between ARM and BRK. (2) ARM can form a bridge between the two DNA binding elements, BRK and TCF, to form a repressing complex *e_1_*TAB*e_2_* (this bridge may involve other intermediate molecules). (3) BRK and dTCF directly associate with each other. For each model, we examine the behavior with and without formation of nonproductive complexes (NPCs) that compete (squelch) nonlinearly with the formation of the repressing complex [2,3]. To model autoactivation of *wg*, we define a functional activation complex as ARM**⋅**TCF bound to *e3*. The values of and references for the parameters used are given in Table 1. Average values are used for unknown parameters. We define a cooperative interaction to be the final step in the formation of a tripartite complex where 2 parts are already bound facilitating the formation of the last interaction. For example, if A•T is bound at *e_1_* and B is bound at *e_2_*, this will facilitate the interaction between A and B to form the functional complex in Case 2. The association rates for the cooperative interactions are ten times faster than normal interactions. The binding reactions for activation and repression under models 1 and 2 are described in Fig. S2. The full set of equations used to describe these are presented in Fig. S3 and the parameters used are described in Table I. Binding reactions and equations for repression model 3 (direct T•B binding) are shown in Fig. S4 and S5 respectively.

Results:

*Exploring three possible modes of repression by TCF, ARM and BRK*

We tested 3 different scenarios for repression. We first explored a concurrent binding model in which A•T and B sites are occupied simultaneously but with no physical interaction between B and the A•T complex (Fig. S1B). If all components are allowed to interact in any order with no bias, this model is described by eleven non-linear ODEs (Fig. S2; S3). To mimic the *in vivo* over-expression of A, B, and T respectively, the formation of a functional repression complex is plotted for 3 cases: when *V_A_* is varied from 10^-7^ to 10^-1^ μM/s, when *V_B_* is varied from10^-4^ to 10^-1^ μM/s and when *V_T_* is varied from 10^-5^ to 10^-3^ μM/s. In agreement with experimental observation, this case predicts that the amount of functional repression complex formed increases with increasing *V_B_* (Fig. S1De), while the amount of repression complex is predicted to decrease with increasing *V_T_* (Fig. S1Df).

Next, we explored a scenario where there is physical contact between T bound at *e_1_* and B bound at *e_2_* that involves a bridge which includes A to form a functional repression complex (Fig. S1). This model demands that A has 2 protein binding surfaces (namely A binds T (directly as is known) and B (either directly or via intermediates). Changes in the relative amounts of A, B, and T determine whether productive repression complexes or non-productive complexes form. An example of the type of non-productive complex that can form is a complex where AT is bound at *e_1_* and AB is bound at *e_2_*, thus preventing the formation of the bridge between T and B (Fig. 6Ci). This system is described by 27 ODEs (Fig. S2; S3). If non-productive complexes are excluded from the system, repression increases with increasing *V_A_* (10^-7^–10^-4^ μM/s), *V_B_* (10^-4^–10^-1^) (Fig. S1Db) but decreases with increasing *V_T_* (10^-5^ to 10^-3^ μM/s; Fig. S1Dj). When non-productive complexes are included in the system, repression complex formation increases at low values of *V_A_* (10^-7^–10^-4^ μM/s) and decreases at higher values (10^-4^–10^-1^ μM/s) as observed experimentally. In addition, repression is still directly related to changes in *V_B_* and inversely related to changes in *V_T_*. Thus, the bridging model (Fig. S1Bii2) with NPCs included is consistent with all experimental observations.

We also explored a third scenario where T and B interact with each other directly and A still binds to T (Fig. S4; S5). NPCs can form in this system too (Fig. S1Biii3), for example A•T can bind at *e_1_* and free T can bind to B at *e_2_* thus preventing the interaction of DNA bound T and B. When non-productive complexes are included, repression complex formation is directly related to changes in A (Fig. S1Dd) and B (Fig. S1Dh) and inversely related to changes in T (Fig. S1Dl). Thus, this model mimics *in vivo* observations for changes in T and B but is inconsistent with the changes observed when A is over-expressed.

The results obtained from exploring these different scenarios suggest that formation of NPCs is a crucial component of this system’s behavior and thus, we focus on the T•A•B bridging model (Fig. S1Bii2) with formation of NPCs in the analyses below.

*Modeling of wg activation by TCF and ARM*

Modeling of *wg* expression in the ventral leg disc in response to WG signaling requires that an ARM•TCF complex bind to the *wg* DNA enhancer binding site *e_3_*. Thus, *wg* expression is a reflection of the formation of the AT*e_3_* complex. The possible interactions involved in formation of AT*e_3_* and the corresponding governing equations are shown in Fig. S3; S3). The computational analysis agrees with the experimental result that over-expression of ARM promotes higher level of *wg* expression. The dashed curves in Fig. S1Da-d show that increasing ARM production rate also increases computationally predicted *wg* expression to a plateau at 100% activation, which is consistent with the experimental observation. In contrast, increasing the production rate of T causes a decrease in the formation of AT*e_3_*, a functioning activation complex. Again this mimics experimental observations. Thus the computational model mimics the effects of excess ARM and TCF on *wg* expression

### Discussion

### Computational modeling can provide a powerful complement to experimental manipulations that can inform and complement our understanding of biological observations. Modeling of the cytosolic events triggered by Wnt signaling has been used to reveal mechanisms of signal transduction and to identify critical targets that regulate the system and thus represent potentially excellent targets for therapeutic intervention [8]. Here we focus on events inside the nucleus, specifically the less well understood process of Wg-dependent repression.

###

### An ARM bridging model faithfully accounts for all aspects of the system behavior

Manipulations of ARM, TCF and BRK levels *in vivo* elicit different changes in *wg* and *dpp* expression. Of the 3 models tested, only the bridging model mimics all the experimental observations (Fig. S1). The effect of altered levels of TCF on *dpp* repression are quite different in the different models. Model (1) and (2) without NPCs show that level of *dpp* repression is insensitive to changes in TCF concentration (Fig. S1Di, j) while both the bridging model with NPCs (model 2) and model (3) show the same trend of decreasing repression with increasing TCF as is observed experimentally. In contrast and in agreement with experimental observation, both *wg* activation and *dpp* repression are relatively insensitive to changing levels of BRK under all scenarios (Fig. S1De-h). The response to altered levels of ARM is the key distinguishing feature among the mechanistic models. We conclude that the response of *dpp* repression to altered levels of A, T and B suggests a mechanism which involves the bridging of ARM between dTCF and BRK which bind to *e_1_* and *e_2_* respectively and the formation of NPCs.

### The results obtained from exploring these scenarios suggest that the formation of NPCs is a critical component of the system’s behavior. These complexes provide a mechanism for squelching by over-expression of a non-DNA binding protein [3].

dTCF is directly required for activation of WG targets (*e.g*. Ubx; [9]) and for the default repression of genes in the absence of WG. Counter to expectation, excess dTCF interferes with WG autoactivation in the ventral leg (Fig. S1Dl). Modeling suggests that as the amount of free T increases, the ratio of T to AT increases such that there is a greater likelihood of forming T*e_3_* than A•T*e_3_* complexes. Excess T also interferes with WG directed repression. As described above, it causes the formation of non-productive repression complexes (Fig. S1Ci) that leads to loss of *dpp* repression (Fig. S1Dk), which in turn antagonizes *wg* activation. Although the response to altered dTCF levels is similar for several of the models, the faithful prediction of this non-intuitive behavior further validates the model.

### The effect of altered BRK levels

Unlike TCF, the response of the system to changes in levels of BRK is much is slower. Analysis suggests that any dominant negative effect in response to elevated BRK would require considerably higher levels of BRK than TCF. Why do excess TCF and BRK levels predict different outcomes? Increasing TCF reduces AT*e_3_* formation. Decreased AT*e_3_* correlates with decreased WG signaling which leads to increased ARM degradation. Thus as T increases, A decreases causing the T:A ratio to rapidly increase (Fig. S6). With a large excess of free T, there is a greater chance that free T rather than AT will bind at *e_3_*. In contrast, increasing BRK production rate does not feed back on ARM degradation, thus the B:A ratio does not show a measurable change over the range of BRK production tested. Thus the relative concentrations of A, B and T are such that as B increases, more productive complexes can form on *e_1_e_2_*. For instance, an increase of *V_T_* from 10^-4^ to 10^-3^ µM decreases the ratio A to T by two orders of magnitude (0.0249 to 0.00065). However, varying *V_B_* by the same value only results a decrease of the same order of magnitude in the A to B ratio (from 93.2 to 9.318). This inter-related chain of interactions results in a close to normal ARM to BRK ratio. Thus, while the squelching effects of excess dTCF affect both *wg* and *dpp* and lead to an increasing cascade of ARM lowering events, the response to increasing BRK leads to a dampening effect that is consistent with the observation that demonstrable changes in the level of repression are not observed in tissues in response to levels of elevated BRK that are achievable experimentally.

### Summary

Our interpretation of experimental results and testing by computational modeling, suggest the following. (1) A two component system with one DNA binding element (e.g.T) and one non-DNA binding element (e.g. A), such as the ARM•dTCF system that activates *wg*, behaves monotonically with respect to altered concentrations of the non-DNA binding component. The ratio of the nonDNA binding to DNA binding components (A:T) is important. At any fixed concentration of T, increasing A, increases wg activation (AT*e_3_*), which further increases A due to feedback. However, when T is increased at a fixed concentration of A, active DNA bound complexes increase until the T:A ratio causes free T to compete with AT for DNA binding, at which point AT*e_3_* formation and thus *wg* activation, decreases.

A system that involves 2 distinct DNA binding components, such as the repression system for *dpp*, can be similarly analyzed. If the two DNA binding components act independently or if they physically interact directly with each other, the level of productive complex on the DNA behaves monotonically with respect to changes in the non-DNA binding component concentration. On the other hand, if there is physical interaction between the two DNA binding components that is mediated through a non-DNA binding component (e.g. such as ARM and possibly additional components) the system reflects a bimodal response to changes in the concentration of the non-DNA binding component(s). This leads to a self-correcting tendency of the system in response to changing levels of the bridging elements (e.g. ARM). Our experimental manipulations demonstrate that a robust self organizing system of morphogen regulation is operative in leg imaginal discs and the theoretical explorations described here support the view that the three component system involving two DNA binding elements interacting with a non-DNA-binding component (BRK•ARM•dTCF) is unique in accounting for the observed behavior of the system to experimental manipulation.

**Supporting Information: Figure legends**

**Figure S1. Computational analysis activation/repression responses of *wg* and *dpp* under different possible modes of action**

**A:** Cartoon key for the 3 proteins and DNA binding sites involved. The *wg* enhancer (*e_3_*) serves to activate *wg* expression, while the *dpp* enhancer (*e_1_e_2_*) contains both TCF (*e_1_*) and BRK (*e_2_*) binding sites and is repressed by WG signaling. Both TCF and BRK bind DNA while ARM does not. **B:** (i) Depicts the TCF based activation complex formed at the *wg* enhancer (ii) depicts 3 possible models of complexes involving TCF, BRK and ARM that might contribute to repression. Model 1 requires concurrent binding of an ARM•dTCF complex and BRK but no physical interaction. Model 2 postulates that repression of *dpp* requires a bridge between TCF and BRK that requires ARM (bridging model). Model 3 proposes a direct binding between TCF and BRK. **C**(i) Examples of non-productive complexes that might form in the presence of high levels of A under the bridging model (1) or that might form in the presence of high levels of T in the direct binding model (2) (ii) examples of the possible sequences of binding events under model 1. There are several possible intermediates on the way to productive complexes (AT*e_3_* or *e_1_*TAB*e_2_*). **D:** The system is experimentally manipulated by increasing or decreasing the production rates (*V_T_, V_A_*, or *V_B_*) of T, A, or B. The computationally predicted response of *wg* activation (dashed line) and *dpp* repression (solid line) to changing levels of T, A or B expression is plotted over a wide range of production rates. The experimentally observed response of wild type *dpp* (e) and *wg* (f) expression to increased levels of ARM production (g, h) and TCF production (i, j) is shown in the bottom panels. The qualitative behavior predicted by the computational analysis disagrees with the concurrent binding and direct T•B binding models but is consistent with the bridging model when non-productive complexes are considered.

**Figure S2 All possible protein-protein and protein-DNA interactions for activation of *wg* and repression of *dpp* by models (1) and (2) are shown.**

Cartoons illustrate the interactions in question and the corresponding binding equations are listed to the right. **A.** Reactions leading to activation of *wg* are shown. **B.** Binding reactions for the concurrent binding model (model 1) are shown where the T•A complex does not bind B. C. Additional binding reactions describing events corresponding to the bridging model (model 2) are shown in a dashed box that correlates with equations in Fig. S3. These binding reactions together with those in B comprise the full set of reactions for the bridging model (2) without formation of NPCs.  **D.** The binding reactions shown in the solid-box describe the formation of all possible NPCs. Together with the reactions shown in B and C, they comprise the full set of reactions for the bridging model with non-productive complexes. Transcriptionally active complexes are shown in bold.

**Figure S3. The equations governing activation and repression models (1) and (2) are shown.**

The unboxed, dash-boxed, and solid-boxed equations/terms correspond to the unboxed, dash-boxed, and solid-boxed interactions in Fig. S2. Model 1 (concurrent binding) is described by the set of equations not enclosed in the dashed and solid-boxes. Model 2 (ARM bridging) is described by the full set of equations. Omitting the terms in the solid-box describes the bridging model (2) in the absence of the formation of NPCs.

**Figure S4. All possible protein-protein and protein-DNA interactions for activation of *wg* and repression of *dpp* by the direct binding model (models 3) are shown.**

Several binding reactions in this model are possible intermediates enroute to final complexes and are identical to binding events shown for other models above. A. Describes the *wg* activation reactions as in Fig. S2). B. Describes intermediate reactions that are the same as the concurrent binding reactions. C. Binding reactions unique to the T•B binding model are shown in the dashed box. D. The binding reactions leading to non-productive complexes in the T•B binding scenario are shown in the solid box. Transcriptionally active complexes are shown in bold.

**Figure S5. Equations governing repression by direct T•B binding (model 3) are shown.**

The complete set of equations describes the behavior of the direct T•B binding reactions in Fig. S4 with the inclusion of non-productive complexes. Omitting the terms in the solid-box describes the behavior under this model (3) in the absence of the formation of NPCs.

**Figure S6. Comparison of the response of T and B to increasing production rates.**

Why is the response to increased production rate of T to squelch T mediated regulation while increasing production rate of B has little effect? The lack of a known feedback on production of T leads to rapid change in the T:A ratio while the known feedback loops governing levels of B tend to maintain a steady ratio of B:A.

Table 1 Descriptions, values, and references of parameters used.

| Symbol | Description | Value and unit | Justification |
| --- | --- | --- | --- |
| *l_+_* | DNA-protein association rate |  | Preliminary data from SPR analysis |
| *l-* | DNA-protein dissociation rate |  | Preliminary data from SPR analysis |
| *k_+_* | protein-protein association rate |  | [10] |
| *k-* | protein-protein dissociation rate |  | [10] |
| *V_A_*  *V_T_*  *V_Bmax_*  *V_Bmin_* | production rate of ARM  Production rate of TCF  Maximum production rate of BRK  Minimum production rate of BRK |  | Covers wide range of production where minimum corresponds to endogenous expression and maximum corresponds to over-expression via Gal4 activation |
| *K_Amax_*  *K_Amin_* | maximum and minimum    degradation rates of ARM |  | Covers wide range where maximum degradation results in no accumulation of Arm (no WG signaling) and minimum degradation mimics WG signaling. |
| *K_degB_* | Degradation rate of BRK |  | Rate of degradation computed from production rate and initial value of BRK to achieve steady state |
|  | EC_50_, effective concentration at 50%  (1) for feedback of *wg* activation on degradation of A  (2) for feedback of *dpp* repression on production of B |  | Values of gammas are chosen so that maximal feedback occurs at the halfway point between the maximum and minimum response values. |
| *m* and *n* | Hill coefficients | 1 | Hill coefficient of one is commonly used to allow a plausible rate of transition from maximum to minimum values e.g. [11,12] |
| (*e_1_e_2_*)_0_ | total DNA binding sites for repression |  | Value corresponds to 2 sites /cell. Cell volume from [13] |
| (*e_3_*)_0_ | total DNA binding sites for activation |  | Value corresponds to 2 sites /cell. Cell volume from [13] |
|  | cooperative association and dissociation factors | 10 and 1 | Reflects cooperative interactions occurring 10 fold faster than non cooperative reactions |

Legend: A brief description of each of the parameters used in modeling is given along with range of values used and references that validate those values.

Literature cited:

1. Barolo S, Posakony JW (2002) Three habits of highly effective signaling pathways: principles of transcriptional control by developmental cell signaling. Genes Dev 16: 1167-1181.

2. Gill G, Ptashne M (1988) Negative effect of the transcriptional activator GAL4. Nature 334: 721-724.

3. Levine M, Manley JL (1989) Transcriptional repression of eukaryotic promoters. Cell 59: 405-408.

4. Meyer ME, Gronemeyer H, Turcotte B, Bocquel MT, Tasset D, et al. (1989) Steroid hormone receptors compete for factors that mediate their enhancer function. Cell 57: 433-442.

5. Jazwinska A, Rushlow C, Roth S (1999) The role of brinker in mediating the graded response to Dpp in early Drosophila embryos. Development 126: 3323-3334.

6. Campbell G, Tomlinson A (1999) Transducing the Dpp morphogen gradient in the wing of Drosophila: regulation of Dpp targets by brinker. Cell 96: 553-562.

7. Minami M, Kinoshita N, Kamoshida Y, Tanimoto H, Tabata T (1999) brinker is a target of Dpp in Drosophila that negatively regulates Dpp-dependent genes. Nature 398: 242-246.

8. Lee E, Salic A, Kruger R, Heinrich R, Kirschner MW (2003) The roles of APC and Axin derived from experimental and theoretical analysis of the Wnt pathway. PLoS Biol 1: E10.

9. Riese J, Yu X, Munnerlyn A, Eresh S, Hsu SC, et al. (1997) LEF-1, a nuclear factor coordinating signaling inputs from wingless and decapentaplegic. Cell 88: 777-787.

10. Knapp S, Zamai M, Volpi D, Nardese V, Avanzi N, et al. (2001) Thermodynamics of the high-affinity interaction of TCF4 with beta-catenin. J Mol Biol 306: 1179-1189.

11. Lander AD, Nie Q, Wan FY (2002) Do morphogen gradients arise by diffusion? Dev Cell 2: 785-796.

12. Fall CP, Marland ES, Wagner JM, Tyson JJ (2002) Computational Cell Biology: Springer Verlag.

13. Eldar A, Shilo BZ, Barkai N (2004) Elucidating mechanisms underlying robustness of morphogen gradients. Curr Opin Genet Dev 14: 435-439.
