# Supplementary material for: Wingless Directly Represses DPP Morphogen Expression via an Armadillo/TCF/Brinker Complex
Source: PLoS One. 2007 Jan 3;2(1):e142. doi: 10.1371/journal.pone.0000142 (PMC1764032; doi:10.1371/journal.pone.0000142)
Supplement: Table S1 — (6.76 MB PDF) [file pone.0000142.s002.pdf]

Table 1 Descriptions, values, and references of parameters used.

Symbol	Description	Value and unit	Justification
l_+	DNA-protein association rate	$2.22\times10^{-2}\,\mu M^{-1}\sec^{-1}$	Preliminary data from SPR analysis
l_-	DNA-protein dissociation rate	$4.11\times10^{-5}\sec^{-1}$	Preliminary data from SPR analysis
k_+	protein-protein association rate	$1.01\times10^{-1}\,\mu M^{-1}\sec^{-1}$	[58]
k_-	protein-protein dissociation rate	$8.47\times10^{-4}\sec^{-1}$	[58]
V_A<br>V_T<br>V_{Bmax}<br>V_{Bmin}	production rate of ARM<br>Production rate of TCF<br>Maximum production rate of BRK<br>Minimum production rate of BRK	$10^{-7}-10^{-1}\,\mu M\sec^{-1}$<br>$10^{-5}-10^{-3}\,\mu M\sec^{-1}$<br>$10^{-4}-10^{-1}\,\mu M\sec^{-1}$<br>$10^{-6}-10^{-3}\,\mu M\sec^{-1}$	Covers wide range of production where minimum corresponds to endogenous expression and maximum corresponds to over-expression via Gal4 activation
K_{Amax}<br>K_{Amin}	maximum and minimum degradation rates of ARM	$10^{-2}\sec^{-1}$<br>$10^{-4}\sec^{-1}$	Covers wide range where maximum degradation results in no accumulation of Arm (no WG signaling) and minimum degradation mimics WG signaling.
K_{degB}	Degradation rate of BRK	$10^{-3}\sec^{-1}$	Rate of degradation computed from production rate and initial value of BRK to achieve steady state
γ_A<br>γ_B	EC_{50}, effective concentration at 50%<br>(1) for feedback of *wg* activation on degradation of A<br>(2) for feedback of *dpp* repression on production of B	$10^{-2}-10^{-4}\,\mu M$	Values of gammas are chosen so that maximal feedback occurs at the halfway point between the maximum and minimum response values.
m and *n*	Hill coefficients	1	Hill coefficient of one is commonly used to allow a plausible rate of transition from maximum to minimum values e.g. [59,60]
$(e_1e_2)_0$	total DNA binding sites for repression	$10^{-3}\,\mu M$	Value corresponds to 2 sites /cell. Cell volume from [61]
$(e_3)_0$	total DNA binding sites for activation	$10^{-3}\,\mu M$	Value corresponds to 2 sites /cell. Cell volume from [61]
f_1 and f_2	cooperative association and dissociation factors	10 and 1	Reflects cooperative interactions occurring 10 fold faster than non cooperative reactions

Legend: A brief description of each of the parameters used in modeling is given along with range of values used and references that validate those values.
