# Supplementary figures and images for: Wingless Directly Represses DPP Morphogen Expression via an Armadillo/TCF/Brinker Complex
Source: PLoS One. 2007 Jan 3;2(1):e142. doi: 10.1371/journal.pone.0000142 (PMC1764032; doi:10.1371/journal.pone.0000142)

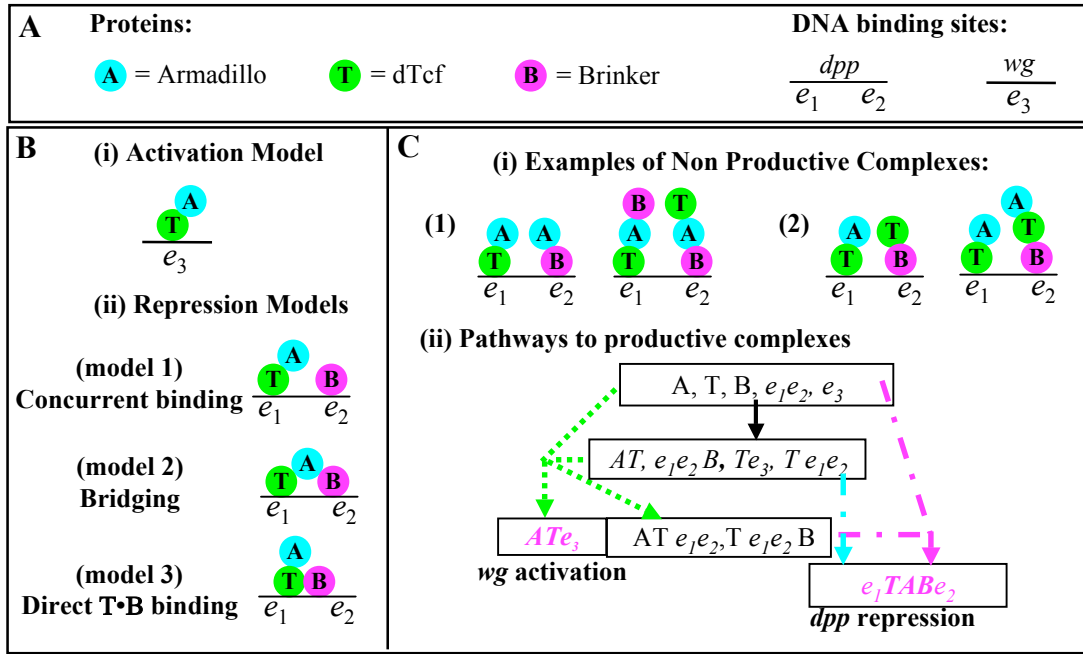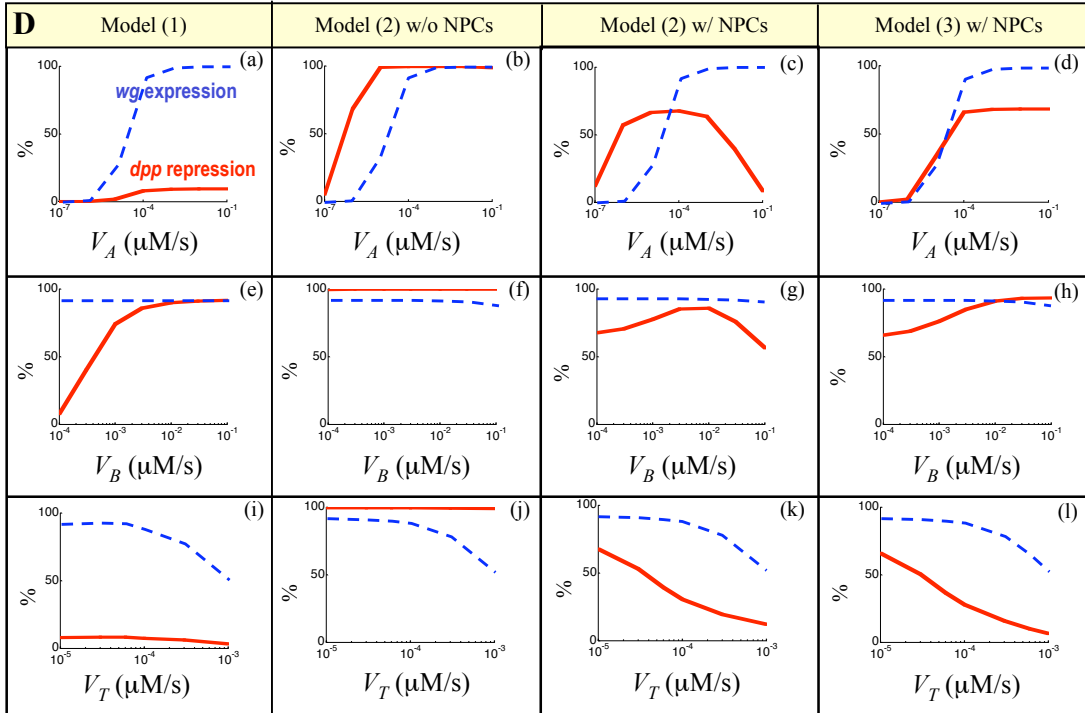

key

— repression  
 - - - activation

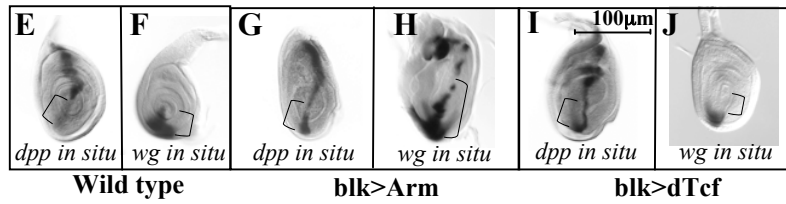

Supplement: Figure S1 — Computational analysis activation/repression responses of wg and dpp under different possible modes of action A: Cartoon key for the 3 proteins and DNA binding sites involved. The wg enhancer (e3) serves to activate wg expression, while the dpp enhancer (e1e2) contains both TCF (e1) and BRK (e2) binding sites and is repressed by WG signaling. Both TCF and BRK bind DNA while ARM does not. B: (i) Depicts the TCF based activation complex formed at the wg enhancer (ii) depicts 3 possible models of complexes involving TCF, BRK and ARM that might contribute to repression. Model 1 requires concurrent binding of an ARM•dTCF complex and BRK but no physical interaction. Model 2 postulates that repression of dpp requires a bridge between TCF and BRK that requires ARM (bridging model). Model 3 proposes a direct binding between TCF and BRK. C(i) Examples of non-productive complexes that might form in the presence of high levels of A under the bridging model (1) or that might form in the presence of high levels of T in the direct binding model (2) (ii) examples of the possible sequences of binding events under model 1. There are several possible intermediates on the way to productive complexes (ATe3 or e1TABe2). D: The system is experimentally manipulated by increasing or decreasing the production rates (VT, VA, or VB) of T, A, or B. The computationally predicted response of wg activation (dashed line) and dpp repression (solid line) to changing levels of T, A or B expression is plotted over a wide range of production rates. The experimentally observed response of wild type dpp (e) and wg (f) expression to increased levels of ARM production (g, h) and TCF production (i, j) is shown in the bottom panels. The qualitative behavior predicted by the computational analysis disagrees with the concurrent binding and direct T•A binding models but is consistent with the bridging model when non-productive complexes are considered. (6.41 MB PDF) [file pone.0000142.s003.pdf]

Fig. S6  
Theisen et. al

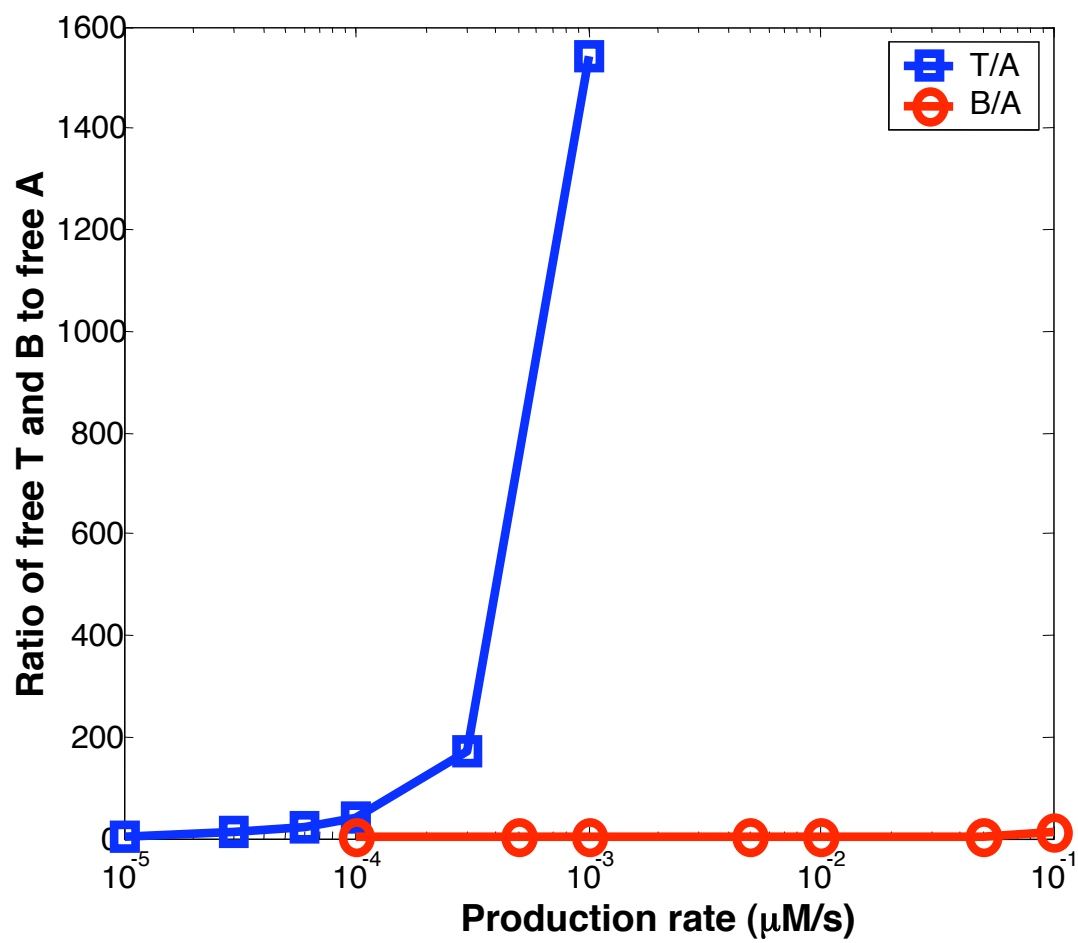

Supplement: Figure S6 — Comparison of the response of T and B to increasing production rates. Why is the response to increased production rate of T to squelch T mediated regulation while increasing production rate of B has little effect? The lack of a known feedback on production of T leads to rapid change in the T:A ratio while the known feedback loops governing levels of B tend to maintain a steady ratio of B:A. (6.24 MB PDF) [file pone.0000142.s008.pdf]
