# Supplementary material for: Wingless Directly Represses DPP Morphogen Expression via an Armadillo/TCF/Brinker Complex
Source: PLoS One. 2007 Jan 3;2(1):e142. doi: 10.1371/journal.pone.0000142 (PMC1764032; doi:10.1371/journal.pone.0000142)

Descriptions of reactions for activation and repression models 1;2 described in Fig S1.

A. *wg* activation reactions (see Fig S1Bi)

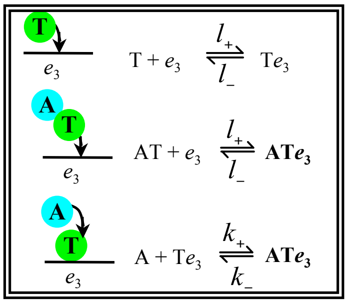

B. Model 1, Concurrent binding reactions (No A-B or T-B binding) (see Fig S1Bii)

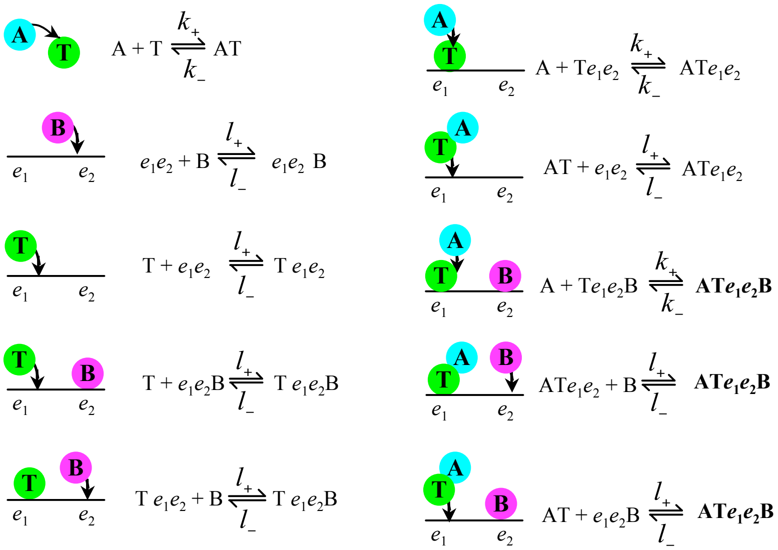

C. Model 2, Reactions leading to productive complexes in ARM bridging model (A-B binding)

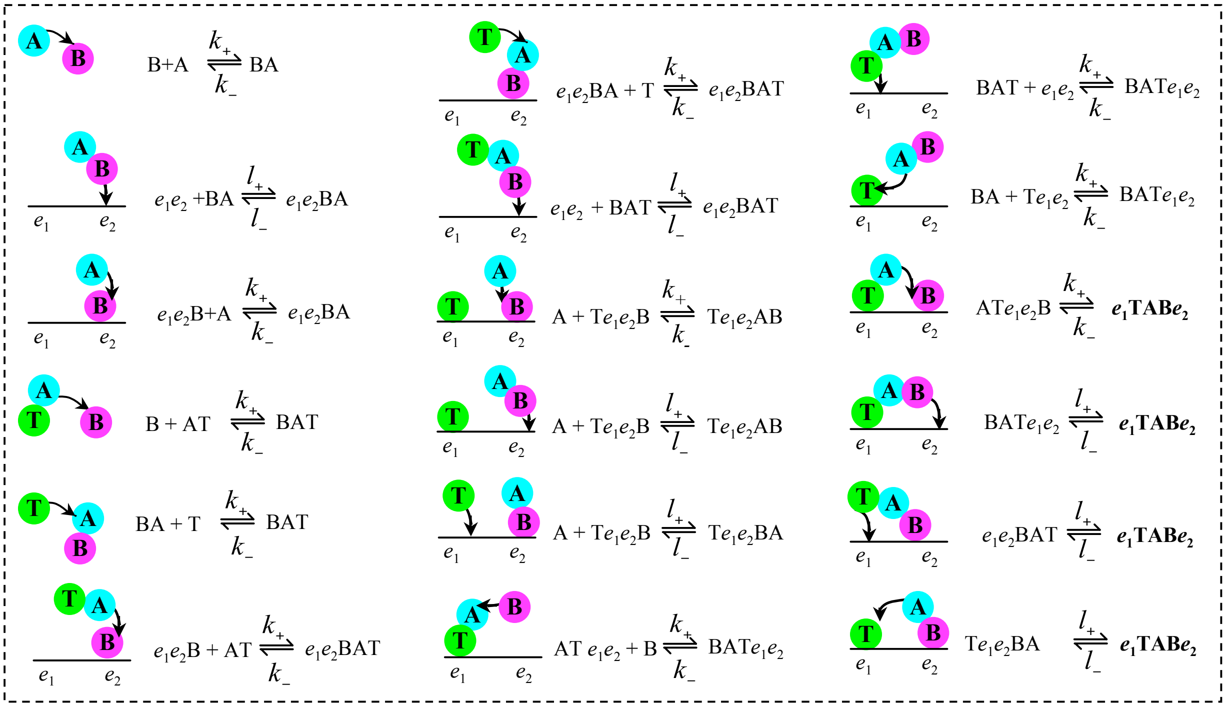

D. Model 2contd: Reactions leading to non-productive complexes in the bridging model (A-B binding)

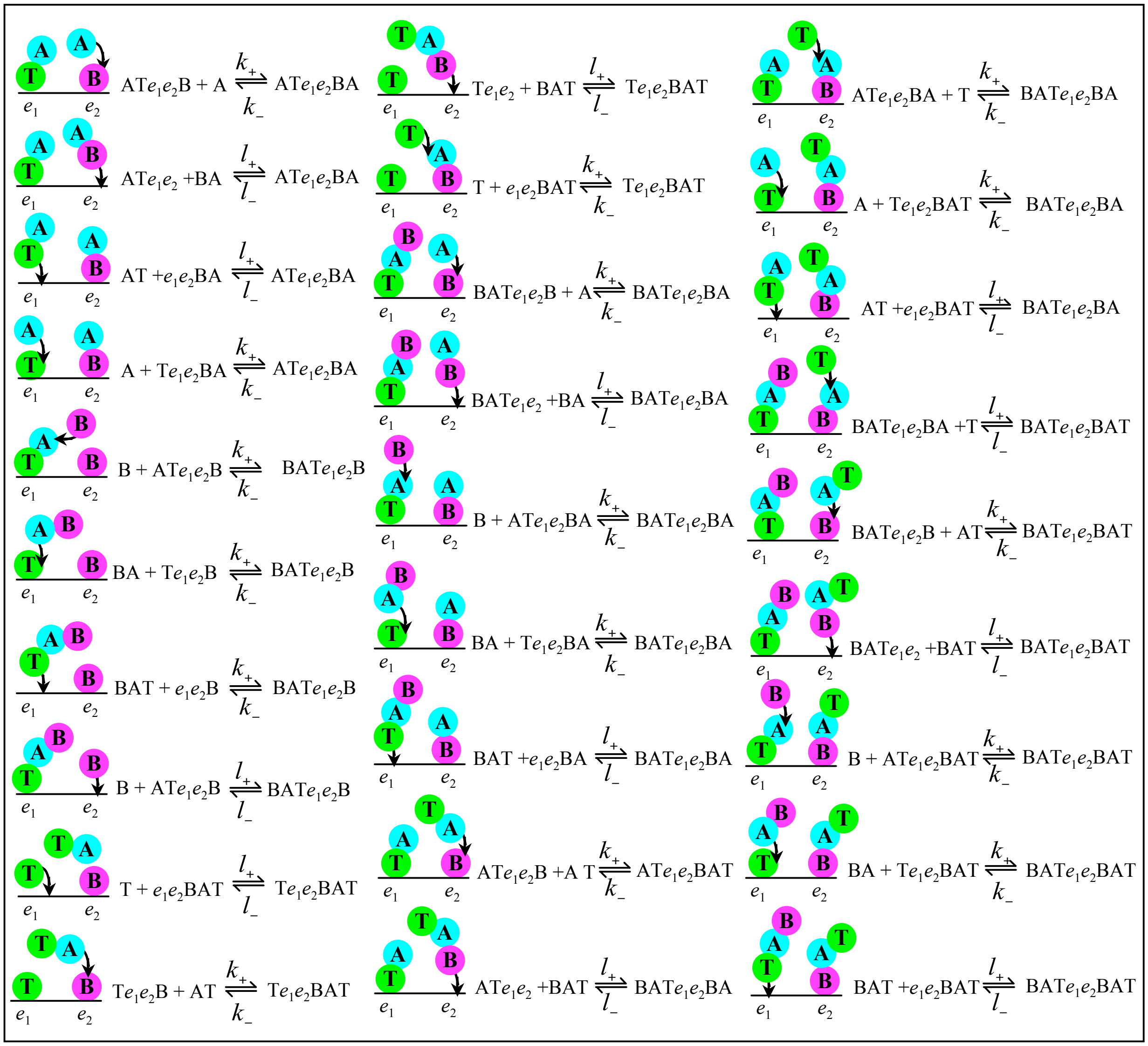

Supplement: Figure S2 — All possible protein-protein and protein-DNA interactions for activation of wg and repression of dpp by models (1) and (2) are shown. Cartoons illustrate the interactions in question and the corresponding binding equations are listed to the right. A. Reactions leading to activation of wg are shown. B. Binding reactions for the concurrent binding model (model 1) are shown where the T•A complex does not bind B. C. Additional binding reactions describing events corresponding to the bridging model (model 2) are shown in a dashed box that correlates with equations in Fig. S3. These binding reactions together with those in B comprise the full set of reactions for the bridging model (2) without formation of NPCs. D. The binding reactions shown in the solid-box describe the formation of all possible NPCs. Together with the reactions shown in B and C, they comprise the full set of reactions for the bridging model with non-productive complexes. Transcriptionally active complexes are shown in bold. (6.24 MB PDF) [file pone.0000142.s004.pdf]
