# Supplementary material for: Wingless Directly Represses DPP Morphogen Expression via an Armadillo/TCF/Brinker Complex
Source: PLoS One. 2007 Jan 3;2(1):e142. doi: 10.1371/journal.pone.0000142 (PMC1764032; doi:10.1371/journal.pone.0000142)
Supplement: Figure S3 — The equations governing activation and repression models (1) and (2) are shown. The unboxed, dash-boxed, and solid-boxed equations/terms correspond to the unboxed, dash-boxed, and solid-boxed interactions in Fig. S2. Model 1 (concurrent binding) is described by the set of equations not enclosed in the dashed and solid-boxes. Model 2 (ARM bridging) is described by the full set of equations. Omitting the terms in the solid-box describes the bridging model (2) in the absence of the formation of NPCs. (6.24 MB PDF) [file pone.0000142.s005.pdf]

Fig. S3
Theisen et. al

Equations for activation and repression models 1&2 described in Fig S1.

(1) $$\frac{d[A]}{dt} = -k_+[A][Te_3] + k_-[ATe_3] - k_+[A][T] + k_-[AT] - k_+[A][Te_1e_2] + k_-[ATe_1e_2] - k_+[A][Te_1e_2B] + k_-[ATe_1e_2B] + V_A - K_{\deg A}[A] +$$
$$-k_+[B][A] + k_-[BA] - k_+[Te_1e_2B][A] + k_-[Te_1e_2BA] - k_+[e_1e_2B][A] + k_-[e_1e_2BA] \quad +$$
$$-k_+[A][Te_1e_2BA] + k_-[ATe_1e_2BA] - k_+[ATe_1e_2B][A] + k_-[ATe_1e_2BA]$$
$$-k_+[BATe_1e_2B][A] + e_1e_2k_-[BATe_1e_2BA] - k_+[A][Te_1e_2BAT] + k_-[ATe_1e_2BAT]$$

(2) $$\frac{d[B]}{dt} = -l_+[ATe_1e_2][B] + l_-[ATe_1e_2B] - l_+[e_1e_2][B] + l_-[e_1e_2B] - l_+[Te_1e_2][B] + l_-[Te_1e_2B] + V_B - K_{\deg B}[B] +$$
$$-k_+[B][A] + k_-[BA] - k_+[B][AT] + k_-[BAT] - k_+[B][ATe_1e_2] + k_-[BATe_1e_2] \quad +$$
$$-l_+[BATe_1e_2][B] + l_-[BATe_1e_2B] - k_+[B][ATe_1e_2BA] + k_-[BATe_1e_2BA]$$
$$-k_+[B][ATe_1e_2BAT] + k_-[BATe_1e_2BAT] - k_+[B][ATe_1e_2B] + k_-[BATe_1e_2B]$$

(3) $$\frac{d[T]}{dt} = -k_+[A][T] + k_-[AT] - l_+[T][e_1e_2] + l_-[Te_1e_2] - l_+[T][e_3] + l_-[Te_3] - l_+[T][e_1e_2B] + l_-[Te_1e_2B] + V_T - K_{\deg T}[T] +$$
$$-k_+[BA][T] + k_-[BAT] - k_+[e_1e_2BA][T] + k_-[e_1e_2BAT] - l_+[T][e_1e_2BA] + l_-[Te_1e_2BA] \quad +$$
$$-l_+[T][e_1e_2BAT] + l_-[Te_1e_2BAT] - k_+[Te_1e_2BA][T] + k_-[Te_1e_2BAT]$$
$$-k_+[ATe_1e_2BA][T] + k_-[ATe_1e_2BAT] - k_+[BATe_1e_2BA][T] + k_-[BATe_1e_2BAT]$$

(4) $$\frac{d[BA]}{dt} = k_+[B][A] - k_-[BA] - k_+[BA][T] + k_-[BAT] - l_+[e_1e_2][BA] + l_-[e_1e_2BA] - k_+[BA][Te_1e_2] + k_-[BAe_1e_2T] - l_+[Te_1e_2][BA] + l_-[Te_1e_2BA] \quad +$$
$$-l_+[ATe_1e_2][BA] + l_-[ATe_1e_2BA] - k_+[BA][Te_1e_2B] + k_-[BATe_1e_2B] - k_+[BA][Te_1e_2BA] + k_-[BATe_1e_2BA]$$
$$-l_+[BATe_1e_2][BA] + l_-[BATe_1e_2BA] - k_+[Te_1e_2BAT][BA] + k_-[BATe_1e_2BAT]$$

(5) $$\frac{d[AT]}{dt} = l_+[A][T] - l_-[AT] - k_+[B][AT] + k_-[BAT] - l_+[AT][e_1e_2] + l_-[ATe_1e_2] - l_+[AT][e_3] + l_-[ATe_3] - l_+[AT][e_1e_2B] + l_-[ATe_1e_2B] +$$
$$-k_+[e_1e_2B][AT] + k_-[e_1e_2BAT] \quad + \quad -l_+[AT][e_1e_2BA] + l_-[ATe_1e_2BA] - k_+[BATe_1e_2B][AT] + k_-[BATe_1e_2BAT] \quad +$$
$$-k_+[Te_1e_2B][AT] + k_-[Te_1e_2BAT] - l_+[AT][e_1e_2BAT] + l_-[ATe_1e_2BAT] - k_+[ATe_1e_2B][AT] + k_-[ATe_1e_2BAT]$$

(6) $$\frac{d[Te_1e_2]}{dt} = l_+[T][e_1e_2] + l_-[Te_1e_2] - k_+A[Te_1e_2] + k_-[ATe_1e_2] - l_+[Te_1e_2][B] + l_-[Te_1e_2B] +$$
$$-k_+[BA][Te_1e_2] + k_-[BATe_1e_2] - l_+[Te_1e_2][BA] + l_-[Te_1e_2BA] \quad + \quad -l_+[Te_1e_2][BAT] + l_-[Te_1e_2BAT]$$

(7) $$\frac{d[e_1e_2B]}{dt} = l_+[e_1e_2][B] - l_-[e_1e_2B] - l_+[T][e_1e_2B] + l_-[Te_1e_2B] - l_+[AT][e_1e_2B] + l_-[ATe_1e_2B] +$$
$$-k_+[e_1e_2B][AT] + k_-[e_1e_2BAT] \quad + \quad -l_+[BAT][e_1e_2B] + l_-[BATe_1e_2B] - k_+[e_1e_2B][A] + k_-[e_1e_2BA]$$

(8) $$\frac{d[Te_3]}{dt} = l_+[T][e_3] + l_-[Te_3] - k_+A[Te_3] + k_-[ATe_3]$$

(9) $$\frac{d[ATe_3]}{dt} = k_+[A][Te_3] - k_-[ATe_3] + l_+[AT][e_3] - l_-[ATe_3]$$

$$
\begin{aligned}
(10)\quad \frac{d[ATe_1e_2]}{dt} = {} & k_+[A][Te_1e_2] - k_-[ATe_1e_2] + l_+[AT][e_1e_2] - l_-[ATe_1e_2] - l_+[ATe_1e_2][B] + l_-[ATe_1e_2B] + \\
& -k_+[B][ATe_1e_2] + k_-[BATe_1e_2] + -l_+[ATe_1e_2][BA] + l_-[ATe_1e_2BA] - l_+[ATe_1e_2][BAT] + l_-[ATe_1e_2BAT]
\end{aligned}
$$

$$
\begin{aligned}
(11)\quad \frac{d[Te_1e_2B]}{dt} = {} & l_+[Te_1e_2][B] - l_-[Te_1e_2B] + l_+[T][e_1e_2B] - l_-[Te_1e_2B] - k_+[A][Te_1e_2B] + k_-[ATe_1e_2B] + \\
& -k_+[Te_1e_2B][A] + k_-[Te_1e_2BA] + -k_+[BA][Te_1e_2B] + k_-[BATe_1e_2B] - k_+[Te_1e_2B][AT] + k_-[Te_1e_2BAT]
\end{aligned}
$$

$$
\begin{aligned}
(12)\quad \frac{d[BAT]}{dt} = {} & k_+[B][AT] - k_-[BAT] + l_+[BA][T] - l_-[BAT] - l_+[BAT][e_1e_2] + l_-[BATe_1e_2] - l_+[e_1e_2][BAT] + l_-[e_1e_2BAT] + \\
& -l_+[Te_1e_2][BAT] + l_-[Te_1e_2BAT] - l_+[BAT][e_1e_2B] + l_-[BATe_1e_2B] - l_+[BAT][e_1e_2BA] + l_-[BATe_1e_2BA] \\
& -l_+[ATe_1e_2][BAT] + l_-[ATe_1e_2BAT] - l_+[BAT][e_1e_2BAT] + l_-[BATe_1e_2BAT] - l_+[BATe_1e_2][BAT] + l_-[BATe_1e_2BAT]
\end{aligned}
$$

$$
\begin{aligned}
(13)\quad \frac{d[e_1e_2BA]}{dt} = {} & l_+[e_1e_2][BA] - l_-[e_1e_2BA] + k_+[e_1e_2B][A] - k_-[e_1e_2BA] - k_+[e_1e_2BA][T] + k_-[e_1e_2BAT] - l_+[T][e_1e_2BA] + l_-[Te_1e_2BA] + \\
& -l_+[AT][e_1e_2BA] + l_-[ATe_1e_2BA] - l_+[BAT][e_1e_2BA] + l_-[BATe_1e_2BA]
\end{aligned}
$$

$$
\begin{aligned}
(14)\quad \frac{d[BATe_1e_2]}{dt} = {} & k_+[B][ATe_1e_2] - k_-[BATe_1e_2] + l_+[BAT][e_1e_2] - l_-[BATe_1e_2] + k_+[BA][Te_1e_2] - k_-[BATe_1e_2] \\
& - f_1 \cdot l_+[BATe_1e_2] + f_2 \cdot l_-[e_1TABe_2] + \\
& -l_+[BATe_1e_2][B] + l_-[BATe_1e_2B] - l_+[BATe_1e_2][BA] + l_-[BATe_1e_2BA]
\end{aligned}
$$

$$
\begin{aligned}
(15)\quad \frac{d[e_1e_2BAT]}{dt} = {} & l_+[e_1e_2][BAT] - l_-[e_1e_2BAT] + k_+[e_1e_2B][AT] - k_-[e_1e_2BAT] + k_+[e_1e_2BA][T] - k_-[e_1e_2BAT] \\
& - f_1 \cdot l_+[e_1e_2BAT] + f_2 \cdot l_-[e_1TABe_2] + \\
& -l_+[T][e_1e_2BAT] + l_-[Te_1e_2BAT] - l_+[AT][e_1e_2BAT] + l_-[ATe_1e_2BAT]
\end{aligned}
$$

$$
\begin{aligned}
(16)\quad \frac{d[Te_1e_2BA]}{dt} = {} & l_+[Te_1e_2][BA] - l_-[Te_1e_2BA] + l_+[T][e_1e_2BA] - l_-[Te_1e_2BA] + k_+[Te_1e_2B][A] - k_-[Te_1e_2BA] \\
& - f_1 \cdot k_+[Te_1e_2BA] + f_2 \cdot k_-[e_1TABe_2] + \\
& -k_+[A][Te_1e_2BA] + k_-[ATe_1e_2BA] - k_+[Te_1e_2BA][T] + k_-[Te_1e_2BAT] - k_+[BA][Te_1e_2BA] + k_-[BATe_1e_2BA]
\end{aligned}
$$

$$
\begin{aligned}
(17)\quad \frac{d[ATe_1e_2B]}{dt} = {} & l_+[AT][e_1e_2B] - l_-[ATe_1e_2B] + k_+[A][Te_1e_2B] - k_-[ATe_1e_2B] + l_+[ATe_1e_2][B] - l_-[ATe_1e_2B] \\
& - f_1 \cdot k_+[ATe_1e_2B] + f_2 \cdot k_-[e_1TABe_2] + \\
& -k_+[ATe_1e_2B][A] + k_-[ATe_1e_2BA] - k_+[B][ATe_1e_2B] + k_-[BATe_1e_2B] - k_+[ATe_1e_2B][AT] + k_-[ATe_1e_2BAT]
\end{aligned}
$$

$$
\begin{aligned}
(18)\quad \frac{d[e_1TABe_2]}{dt} = {} & f_1 \cdot k_+[ATe_1e_2B] - f_2 \cdot k_-[e_1TABe_2] + f_1 \cdot k_+[Te_1e_2BA] - f_2 \cdot k_-[e_1TABe_2] + f_1 \cdot l_+[e_1e_2BAT] + f_2 \cdot l_-[e_1TABe_2] \\
& + f_1 \cdot l_+[BATe_1e_2] - f_2 \cdot l_-[e_1TABe_2]
\end{aligned}
$$

$$(19)\quad \frac{d[ATe_1e_2BA]}{dt} = k_+[A][Te_1e_2BA] - k_-[ATe_1e_2BA] + l_+[AT][e_1e_2BA] - l_-[ATe_1e_2BA] + k_+[ATe_1e_2B][A] - k_-[ATe_1e_2BA] + l_+[ATe_1e_2][BA] - l_-[ATe_1e_2BA] - k_+[B][ATe_1e_2BA] + k_-[BATe_1e_2BA] - k_+[ATe_1e_2BA][T] + k_-[ATe_1e_2BAT]$$

$$(20)\quad \frac{d[BATe_1e_2B]}{dt} = k_+[B][ATe_1e_2B] - k_-[BATe_1e_2B] + k_+[BA][Te_1e_2B] - k_-[BATe_1e_2B] + l_+[BAT][e_1e_2B] - l_-[BATe_1e_2B] + l_+[BATe_1e_2][B] - l_-[BATe_1e_2B] - k_+[BATe_1e_2B][A] + k_-[BATe_1e_2BA]$$

$$(21)\quad \frac{d[Te_1e_2BAT]}{dt} = l_+[T][e_1e_2BAT] - l_-[Te_1e_2BAT] + k_+[Te_1e_2BA][T] - k_-[Te_1e_2BAT] + k_+[Te_1e_2B][AT] - k_-[Te_1e_2BAT] + l_+[Te_1e_2][BAT] - l_-[Te_1e_2BAT] - k_+[A][Te_1e_2BAT] + k_-[ATe_1e_2BAT]$$

$$(22)\quad \frac{d[BATe_1e_2BA]}{dt} = k_+[B][ATe_1e_2BA] - k_-[BATe_1e_2BA] + k_+[BA][Te_1e_2BA] - k_-[BATe_1e_2BA] + l_+[BAT][e_1e_2BA] - \underline{l}[BATe_1e_2BA] + l_+[BATe_1e_2][BA] - l_-[BATe_1e_2BA] + k_+[BATe_1e_2B][A] - k_-[BATe_1e_2BA] - k_+[BATe_1e_2BA][T] + k_-[BATe_1e_2BAT]$$

$$(23)\quad \frac{d[ATe_1e_2BAT]}{dt} = l_+[AT][e_1e_2BAT] - l_-[ATe_1e_2BAT] + k_+[A][Te_1e_2BAT] - k_-[ATe_1e_2BAT] + k_+[ATe_1e_2B][AT] - k_-[ATe_1e_2BAT] + l_+[ATe_1e_2][BAT] - l_-[ATe_1e_2BAT] + k_+[ATe_1e_2BA][T] - k_-[ATe_1e_2BAT] - k_+[B][ATe_1e_2BAT] + k_-[BATe_1e_2BAT]$$

$$(24)\quad \frac{d[BATe_1e_2BAT]}{dt} = l_+[BAT][e_1e_2BAT] - l_-[BATe_1e_2BAT] + l_+[BAT][e_1e_2BAT] - l_-[BATe_1e_2BAT] + k_+[BATe_1e_2B][AT] - k_-[BATe_1e_2BAT] + k_+[BA][Te_1e_2BAT] - k_-[BATe_1e_2BAT] + k_+[B][ATe_1e_2BAT] - k_-[BATe_1e_2BAT] + k_+[BATe_1e_2BA][T] - k_-[BATe_1e_2BAT]$$
