# Supplementary material for: Wingless Directly Represses DPP Morphogen Expression via an Armadillo/TCF/Brinker Complex
Source: PLoS One. 2007 Jan 3;2(1):e142. doi: 10.1371/journal.pone.0000142 (PMC1764032; doi:10.1371/journal.pone.0000142)

Descriptions of reactions involved in repression model 3 (direct T•B binding) described in Fig S1.

A. *wg* activation reactions (same as Fig S1)

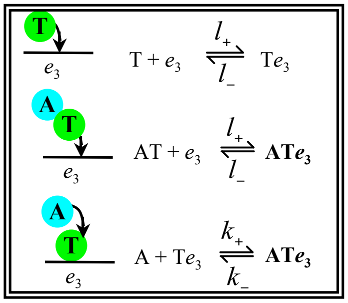

B. Intermediate reactions (same as Model 1, Concurrent binding reactions, Fig S1).

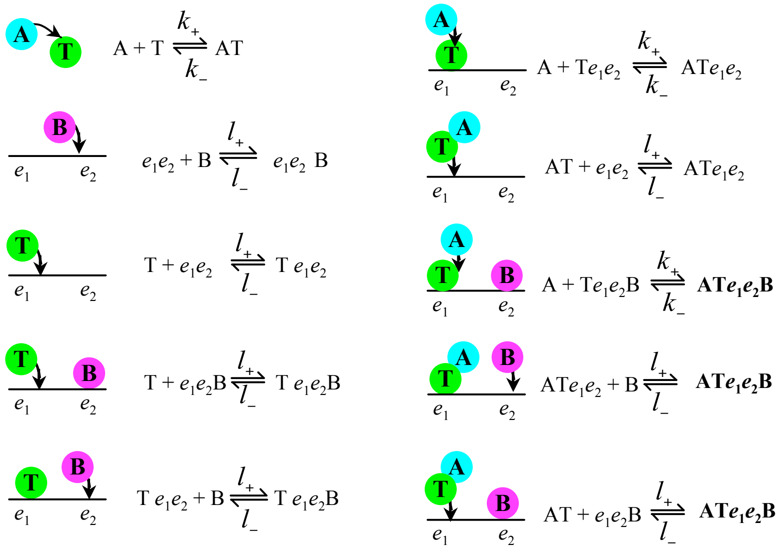

C. Model 3: Reactions leading to productive complexes in the direct T•B binding model

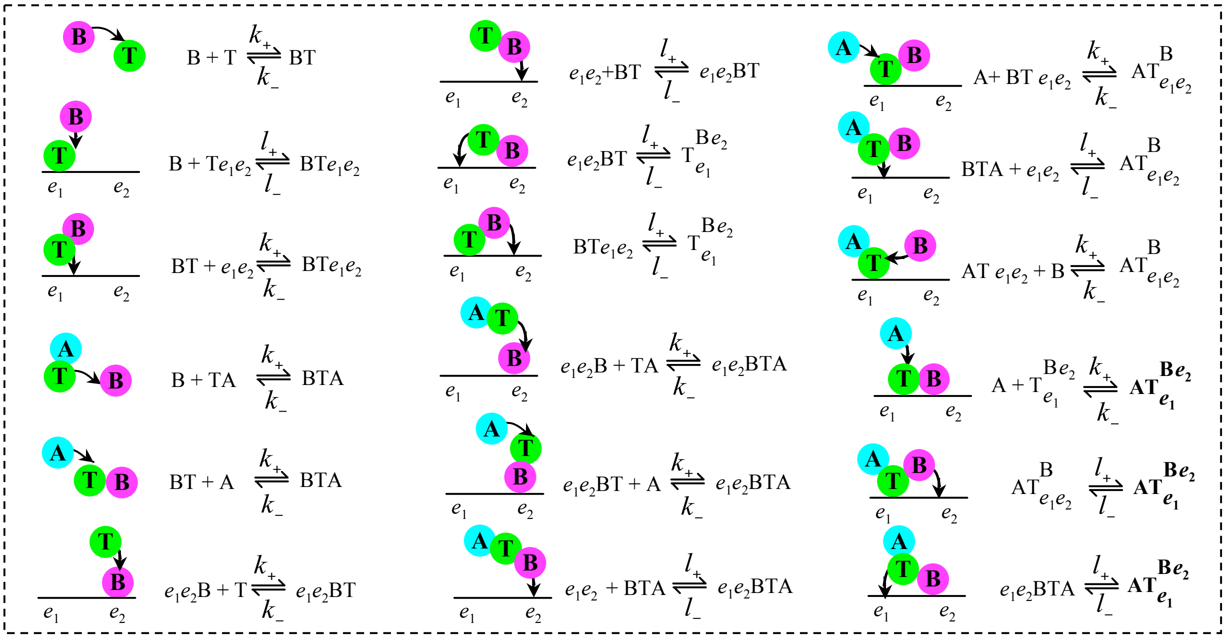

D. Model 3contd: Reactions leading to non-productive complexes in the direct T•B binding model

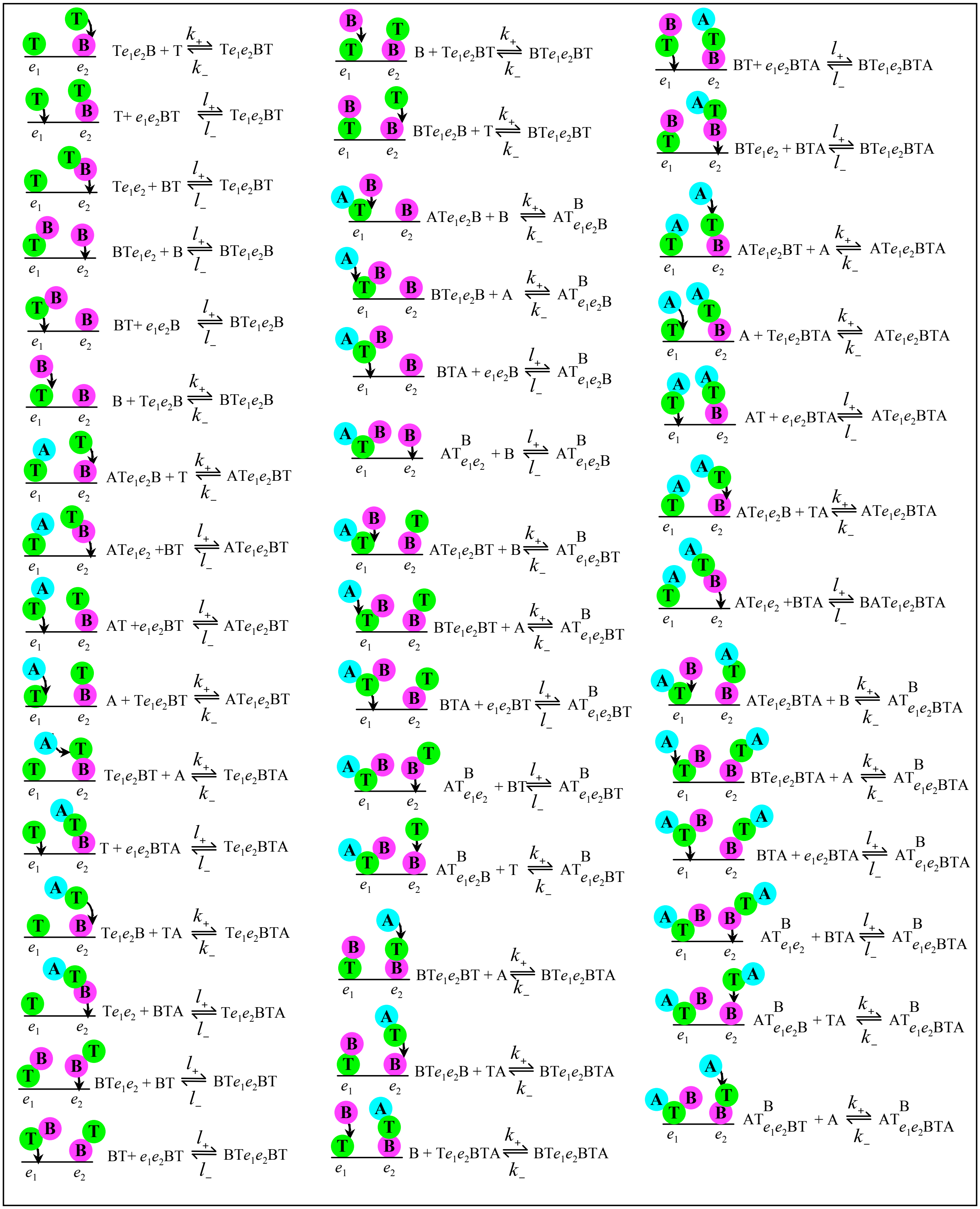

Supplement: Figure S4 — All possible protein-protein and protein-DNA interactions for activation of wg and repression of dpp by the direct binding model (models 3) are shown. Several binding reactions in this model are possible intermediates enroute to final complexes and are identical to binding events shown for other models above. A. Describes the wg activation reactions as in Fig. S2). B. Describes intermediate reactions that are the same as the concurrent binding reactions. C. Binding reactions unique to the T•B binding model are shown in the dashed box. D. The binding reactions leading to non-productive complexes in the T•B binding scenario are shown in the solid box. Transcriptionally active complexes are shown in bold. (6.24 MB PDF) [file pone.0000142.s006.pdf]
