# Supplementary material for: Wingless Directly Represses DPP Morphogen Expression via an Armadillo/TCF/Brinker Complex
Source: PLoS One. 2007 Jan 3;2(1):e142. doi: 10.1371/journal.pone.0000142 (PMC1764032; doi:10.1371/journal.pone.0000142)
Supplement: Figure S5 — Equations governing repression by direct T•B binding (model 3) are shown. The complete set of equations describes the behavior of the direct T•B binding reactions in Fig. S4 with the inclusion of non-productive complexes. Omitting the terms in the solid-box describes the behavior under this model (3) in the absence of the formation of NPCs. (6.24 MB PDF) [file pone.0000142.s007.pdf]

Fig. S5
Theisen et. al

Equations for repression model 3 (direct T•B binding) described in Fig S1.

(1')
$$\frac{d[A]}{dt} = -k_+[A][Te_3] + k_-[ATe_3] - k_+[A][T] + k_-[AT] - k_+[A][Te_1e_2] + k_-[ATe_1e_2] - k_+[A][Te_1e_2B] + k_-[ATe_1e_2B] + V_A - K_{\deg A}[A] +$$
$$- k_+[BT][A] + k_-[BTA] - k_+[A][BTR] + k_-[AT^B_{e_1e_2}] - k_+[RBT][A] + k_-[RBTA] - k_+[A][T^{Be_2}_{e_1}] + k_-[AT^{Be_2}_{e_1}] +$$
$$- k_+[A][Te_1e_2BT] + k_-[ATe_1e_2BT] - k_+[Te_1e_2BT][A] + k_-[Te_1e_2BTA] - k_+[BTe_1e_2BT][A] + k_-[BTe_1e_2BTA]$$
$$- k_+[ATe_1e_2BT][A] + k_-[ATe_1e_2BTA] - k_+[AT^B_{e_1e_2BT}][A] + k_-[AT^B_{e_1e_2BTA}] - k_+[A][BTe_1e_2B] + k_-[AT^B_{e_1e_2B}]$$
$$- k_+[BTe_1e_2BT][A] + k_-[AT^B_{e_1e_2BT}] - k_+[A][Te_1e_2BTA] + k_-[ATe_1e_2BTA]$$

(2')
$$\frac{d[B]}{dt} = -l_+[ATe_1e_2][B] + l_-[ATe_1e_2B] - l_+[e_1e_2][B] + l_-[e_1e_2B] - l_+[Te_1e_2][B] + l_-[Te_1e_2B] + V_B - K_{\deg B}[B] +$$
$$- k_+[B][T] + k_-[BT] - k_+[B][TA] + k_-[BTA] - k_+[B][Te_1e_2] + k_-[BTe_1e_2] - k_+[ATe_1e_2][B] + k_-[AT^B_{e_1e_2}] +$$
$$- k_+[B][Te_1e_2B] + k_-[BTe_1e_2B] - l_+[BTe_1e_2][B] + l_-[BTe_1e_2B] - k_+[B][Te_1e_2BT] + k_-[BTe_1e_2BT] - k_+[B][Te_1e_2BTA] + k_-[BTe_1e_2BTA]$$
$$- k_+[ATe_1e_2BTA][B] + k_-[AT^B_{e_1e_2BTA}] - l_+[AT^B_{e_1e_2}][B] + l_-[AT^B_{e_1e_2B}] - k_+[ATe_1e_2B][B] + k_-[AT^B_{e_1e_2B}] - k_+[ATe_1e_2BT][B] + k_-[AT^B_{e_1e_2BT}]$$

(3')
$$\frac{d[T]}{dt} = -k_+[A][T] + k_-[AT] - l_+[T][e_1e_2] + l_-[Te_1e_2] - l_+[T][e_3] + l_-[Te_3] - l_+[T][e_1e_2B] + l_-[Te_1e_2B] + V_T - K_{\deg T}[T] +$$
$$- k_+[B][T] + k_-[BT] - k_+[e_1e_2B][T] + k_-[e_1e_2BT] +$$
$$- l_+[T][e_1e_2BT] + l_-[Te_1e_2BT] - k_+[Te_1e_2B][T] + k_-[Te_1e_2BT] - l_+[ATe_1e_2B][T] + l_-[ATe_1e_2BT]$$
$$- k_+[BTe_1e_2B][T] + k_-[BTe_1e_2BT] - l_+[T][e_1e_2BTA] + l_-[Te_1e_2BTA] - k_+[AT^B_{e_1e_2B}][T] + k_-[AT^B_{e_1e_2BT}]$$

(4')
$$\frac{d[BT]}{dt} = k_+[B][T] - k_-[BT] - k_+[BT][A] + k_-[BTA] - l_+[BT][e_1e_2] + l_-[BTe_1e_2] - l_+[e_1e_2][BT] + l_-[e_1e_2BT] +$$
$$- l_+[Te_1e_2][BT] + l_-[Te_1e_2BT] - l_+[BT][e_1e_2B] + l_-[BTe_1e_2B] - l_+[ATe_1e_2][BT] + l_-[ATe_1e_2BT] - l_+[BT][e_1e_2BT] + l_-[BTe_1e_2BT]$$
$$- l_+[BTe_1e_2][BT] + l_-[BTe_1e_2BT] - l_+[BT][e_1e_2BTA] + l_-[BTe_1e_2BTA] - l_+[AT^B_{e_1e_2}][BT] + l_-[AT^B_{e_1e_2BT}]$$

(5')
$$\frac{d[AT]}{dt} = k_+[A][T] - k_-[AT] - l_+[AT][e_1e_2] + l_-[ATe_1e_2] - l_+[AT][e_3] + l_-[ATe_3] - l_+[AT][e_1e_2B] + l_-[ATe_1e_2B] +$$
$$- k_+[B][AT] + k_-[BTA] - k_+[e_1e_2B][AT] + k_-[e_1e_2BTA] +$$
$$- l_+[AT][e_1e_2BT] + l_-[ATe_1e_2BT] - k_+[Te_1e_2B][AT] + k_-[Te_1e_2BTA] - k_+[BTe_1e_2B][AT] + k_-[BTe_1e_2BTA]$$
$$- l_+[AT][e_1e_2BTA] + l_-[ATe_1e_2BTA] - k_+[ATe_1e_2B][AT] + k_-[ATe_1e_2BTA] - k_+[AT^B_{e_1e_2B}][AT] + k_-[AT^B_{e_1e_2BTA}]$$

(6')
$$\frac{d[Te_1e_2]}{dt} = l_+[T][e_1e_2] + l_-[Te_1e_2] - k_+A[Te_1e_2] + k_-[ATe_1e_2] - l_+[Te_1e_2][B] + l_-[Te_1e_2B] +$$
$$- k_+[B][Te_1e_2] + k_-[BTe_1e_2] + - l_+[Te_1e_2][BT] + l_-[Te_1e_2BT] - l_+[Te_1e_2][BTA] + l_-[Te_1e_2BTA]$$

(7')
$$\frac{d[e_1e_2B]}{dt} = l_+[e_1e_2][B] - l_-[e_1e_2B] - l_+[T][e_1e_2B] + l_-[Te_1e_2B] - l_+[AT][e_1e_2B] + l_-[ATe_1e_2B] +$$
$$- k_+[e_1e_2B][T] + k_-[e_1e_2BT] - k_+[e_1e_2B][TA] + k_-[e_1e_2BTA] + - l_+[BT][e_1e_2B] + l_-[BTe_1e_2B] - l_+[BTA][e_1e_2B] + l_-[AT^B_{e_1e_2B}]$$

(8')
$$\frac{d[Te_3]}{dt} = l_+[T][e_3] + l_-[Te_3] - k_+A[Te_3] + k_-[ATe_3]$$

(9')
$$\frac{d[ATe_3]}{dt} = k_+[A][Te_3] - k_-[ATe_3] + l_+[AT][e_3] - l_-[ATe_3]$$

$$(10')\ \frac{d[ATe_1e_2]}{dt} = k_+[A][Te_1e_2] - k_-[ATe_1e_2] + l_+[AT][e_1e_2] - l_-[ATe_1e_2] - l_+[ATe_1e_2][B] + l_-[ATe_1e_2B] +$$
$$- k_+[ATe_1e_2][B] + k_-[AT_{e_1e_2}^{B}] + - l_+[ATe_1e_2][BT] + l_-[ATe_1e_2BT] - l_+[ATe_1e_2][BTA] + l_-[ATe_1e_2BTA]$$

$$(11')\ \frac{d[Te_1e_2B]}{dt} = l_+[Te_1e_2][B] - l_-[Te_1e_2B] + l_+[T][e_1e_2B] - l_-[Te_1e_2B] - k_+[A][Te_1e_2B] + k_-[ATe_1e_2B] +$$
$$- k_+[Te_1e_2B][A] + k_-[Te_1e_2BA] + - k_+[Te_1e_2B][T] + k_-[Te_1e_2BT] - k_+[B][Te_1e_2B] + k_-[BTe_1e_2B] - k_+[Te_1e_2B][AT + k_-[Te_1e_2BTA]$$

$$(12')\ \frac{d[BTA]}{dt} = k_+[B][TA] - k_-[BTA] + l_+[BT][A] - l_-[BTA] - l_+[BTA][e_1e_2] + l_-[AT_{e_1e_2}^{B}]$$
$$- l_+[e_1e_2][BTA] + l_-[e_1e_2BTA] - l_+[e_1e_2][BTA] + l_-[AT_{e_1}^{Be_2}] +$$
$$- l_+[Te_1e_2][BTA] + l_-[Te_1e_2BTA] - l_+[ATe_1e_2][BTA] + l_-[ATe_1e_2BTA] - l_+[BTA][e_1e_2B] + l_-[AT_{e_1e_2B}^{B}] - l_+[BTA][e_1e_2BT]$$
$$+ l_-[AT_{e_1e_2BT}^{B}] - l_+[AT_{e_1e_2}^{B}][BTA] + l_-[AT_{e_1e_2BTA}^{B}] - l_+[BTA][e_1e_2BTA] + l_-[AT_{e_1e_2BTA}^{B}] - l_+[BTe_1e_2][BTA] + l_-[BTe_1e_2BTA]$$

$$(13')\ \frac{d[e_1e_2BT]}{dt} = l_+[e_1e_2][BT] - l_-[e_1e_2BT] + k_+[e_1e_2B][T] - k_-[e_1e_2BT] - l_+[e_1e_2BT] + l_-[T_{e_1}^{Be_2}] - l_+[e_1e_2BT][A] + l_-[e_1e_2BTA] +$$
$$- l_+[T][e_1e_2BT] + l_-[Te_1e_2BT] - l_+[AT][e_1e_2BT] + l_-[ATe_1e_2BT] - l_+[BT][e_1e_2BT] + l_-[BTe_1e_2BT] - l_+[BTA][e_1e_2BT] + l_-[AT_{e_1e_2BT}^{B}]$$

$$(14')\ \frac{d[BTe_1e_2]}{dt} = l_+[BT][e_1e_2] - l_-[BTe_1e_2] + k_+[B][Te_1e_2] - k_-[BTe_1e_2] - l_+[BTe_1e_2] + l_-[T_{e_1}^{Be_2}] - k_+[A][BTe_1e_2] + k_-[AT_{e_1e_2}^{B}] +$$
$$- l_+[BTe_1e_2][B] + l_-[BTe_1e_2B] - l_+[BTe_1e_2][BT] + l_-[BTe_1e_2BT] - l_+[BTe_1e_2][BTA] + l_-[BTe_1e_2BTA]$$

$$(15')\ \frac{d[T_{e_1}^{Be_2}]}{dt} = l_+[BTe_1e_2] - l_-[T_{e_1}^{Be_2}] + l_+[e_1e_2BT] - l_-[T_{e_1}^{Be_2}] - k_+[A][T_{e_1}^{Be_2}] + k_-[AT_{e_1}^{Be_2}]$$

$$(16')\ \frac{d[AT_{e_1e_2}^{B}]}{dt} = k_+[ATe_1e_2][B] - k_-[AT_{e_1e_2}^{B}] + l_+[BTA][e_1e_2] - l_-[AT_{e_1e_2}^{B}] + k_+[A][BTe_1e_2] - k_-[AT_{e_1e_2}^{B}]$$
$$- l_+[AT_{e_1e_2}^{B}][B] + l_-[AT_{e_1e_2B}^{B}] - l_+[AT_{e_1e_2}^{B}][BT] + l_-[AT_{e_1e_2BT}^{B}] - l_+[AT_{e_1e_2}^{B}][BTA] + l_-[AT_{e_1e_2BTA}^{B}]$$
$$- l_+[AT_{e_1e_2}^{B}][BT] + l_-[AT_{e_1e_2BT}^{B}] - l_+[AT_{e_1e_2}^{B}][BTA] + l_-[AT_{e_1e_2BTA}^{B}]$$

$$(17')\ \frac{d[e_1e_2BTA]}{dt} = l_+[e_1e_2][BTA] - l_-[e_1e_2BTA] + k_+[e_1e_2B][AT] - k_-[e_1e_2BTA] + k_+[e_1e_2BT][A] - k_-[e_1e_2BTA]$$
$$- f_1 \cdot l_+[e_1e_2BTA] + f_2 \cdot l_-[AT_{e_1}^{Be_2}] +$$
$$- l_+[T][e_1e_2BTA] + l_-[Te_1e_2BTA] - l_+[BT][e_1e_2BTA] + l_-[BTe_1e_2BTA] - l_+[AT][e_1e_2BTA] + l_-[ATe_1e_2BTA]$$
$$- l_+[BTA][e_1e_2BTA] + l_-[AT_{e_1e_2BTA}^{B}]$$

$$(18')\ \frac{d[ATe_1e_2B]}{dt} = l_+[AT][e_2e_2B] - l_-[ATe_1e_2B] + k_+[A][Te_1e_2B] - k_-[ATe_1e_2B] + l_+[ATe_1e_2][B] - l_-[ATe_1e_2B]$$
$$- k_+[ATe_1e_2B][A] + k_-[ATe_1e_2BA] - k_+[ATe_1e_2B][AT] + k_-[ATe_1e_2BTA] - k_+[ATe_1e_2B][B] + k_-[AT_{e_1e_2B}^{B}]$$

$$(19')\ \frac{d[AT_{e_1}^{Be_2}]}{dt} = k_+[A][T_{e_1}^{Be_2}] - k_-[AT_{e_1}^{Be_2}] + k_+[e_1e_2][BTA] - k_-[AT_{e_1}^{Be_2}] + f_1 \cdot l_+[e_1e_2BTA] + f_2 \cdot l_-[AT_{e_1}^{Be_2}]$$

(20’)
$$\frac{d[Te_1e_2BT]}{dt} = l_+[T][e_1e_2BT] - l_-[Te_1e_2BT] + l_+[Te_1e_2][BT] - l_-[Te_1e_2BT] + k_+[Te_1e_2B][T] - k_-[Te_1e_2BT] - k_+[A][Te_1e_2BT] + k_-[ATe_1e_2BT] - l_+[B][Te_1e_2BT] + l_-[BTe_1e_2BT] - k_+[Te_1e_2BT][A] + k_-[Te_1e_2BTA]$$

(21’)
$$\frac{d[BTe_1e_2B]}{dt} = k_+[B][Te_1e_2B] - k_-[BTe_1e_2B] + l_+[BT][e_1e_2B] - l_-[BTe_1e_2B] - k_+[BTe_1e_2B][T] + k_-[BTe_1e_2BT] + l_+[BTe_1e_2][B] - l_-[BTe_1e_2B] - k_+[BTe_1e_2B][AT] + k_-[Te_1e_2BTA] - k_+[A][BTe_1e_2B] + k_-[AT^B_{e_1e_2B}]$$

(22’)
$$\frac{d[ATe_1e_2BT]}{dt} = k_+[A][Te_1e_2BT] - k_-[ATe_1e_2BT] + l_+[AT][e_1e_2BT] - l_-[ATe_1e_2BT] + k_+[ATe_1e_2B][T] - k_-[ATe_1e_2BT] + l_+[ATe_1e_2][BT] - l_-[ATe_1e_2BT] - k_+[ATe_1e_2BT][A] + k_-[ATe_1e_2BTA] - k_+[ATe_1e_2BT][B] + k_-[AT^B_{e_1e_2BT}]$$

(23’)
$$\frac{d[BTe_1e_2BT]}{dt} = k_+[B][Te_1e_2BT] - k_-[BTe_1e_2BT] + l_+[BT][e_1e_2BT] - l_-[BTe_1e_2BT] + k_+[BTe_1e_2B][T] - k_-[BTe_1e_2BT] + l_+[BTe_1e_2][BT] - l_-[BTe_1e_2BT] - k_+[BTe_1e_2BT][A] + k_-[Te_1e_2BTA] - k_+[A][BTe_1e_2BT] + k_-[AT^B_{e_1e_2BT}]$$

(24’)
$$\frac{d[Te_1e_2BTA]}{dt} = k_+[Te_1e_2BT][A] - k_-[Te_1e_2BTA] + l_+[T][e_1e_2BTA] - l_-[Te_1e_2BTA] + k_+[Te_1e_2B][AT] - k_-[Te_1e_2BTA] + l_+[Te_1e_2][BTA] - l_-[Te_1e_2BTA] - k_+[B][Te_1e_2BTA] + k_-[BTe_1e_2BTA] - k_+[A][Te_1e_2BTA] + k_-[ATe_1e_2BTA]$$

(25’)
$$\frac{d[BTe_1e_2BTA]}{dt} = k_+[B][Te_1e_2BTA] - k_-[BTe_1e_2BTA] + l_+[BT][e_1e_2BTA] - l_-[BTe_1e_2BTA] + l_+[BTe_1e_2][BTA] - l_-[BTe_1e_2BTA] + k_+[BTe_1e_2BT][A] - k_-[BTe_1e_2BTA] + k_+[BTe_1e_2B][AT] - k_-[BTe_1e_2BTA] - k_+[A][BTe_1e_2BTA] + k_-[AT^B_{e_1e_2BTA}]$$

(26’)
$$\frac{d[ATe_1e_2BTA]}{dt} = k_+[A][Te_1e_2BTA] - k_-[ATe_1e_2BTA] + l_+[AT][e_1e_2BTA] - l_-[ATe_1e_2BTA] + l_+[ATe_1e_2][BTA] - l_-[ATe_1e_2BTA] + k_+[ATe_1e_2B][AT] - k_-[ATe_1e_2BTA] - k_+[ATe_1e_2BTA][B] + k_-[AT^B_{e_1e_2BTA}]$$

(27’)
$$\frac{d[AT^B_{e_1e_2B}]}{dt} = k_+[A][BTe_1e_2B] - k_-[AT^B_{e_1e_2B}] + l_+[BTA][e_1e_2B] - l_-[AT^B_{e_1e_2B}] + l_+[AT^B_{e_1e_2}][B] - l_-[AT^B_{e_1e_2B}] + k_+[ATe_1e_2B][B] - k_-[AT^B_{e_1e_2B}] - k_+[AT^B_{e_1e_2B}][T] + k_-[AT^B_{e_1e_2BT}] - k_+[AT^B_{e_1e_2B}][AT] + k_-[AT^B_{e_1e_2BTA}]$$

(28’)
$$\frac{d[AT^B_{e_1e_2BT}]}{dt} = k_+[A][BTe_1e_2BT] - k_-[AT^B_{e_1e_2BT}] + l_+[BTA][e_1e_2BT] - l_-[AT^B_{e_1e_2BT}] + l_+[AT^B_{e_1e_2B}][T] - l_-[AT^B_{e_1e_2BT}] + k_+[ATe_1e_2BT][B] - k_-[AT^B_{e_1e_2BT}] + l_+[AT^B_{e_1e_2}][BT] - l_-[AT^B_{e_1e_2BT}] - k_+[AT^B_{e_1e_2BT}][A] + k_-[AT^B_{e_1e_2BTA}]$$

(29’)
$$\frac{d[AT^B_{e_1e_2BTA}]}{dt} = k_+[A][BTe_1e_2BTA] - k_-[AT^B_{e_1e_2BTA}] + l_+[BTA][e_1e_2BTA] - l_-[AT^B_{e_1e_2BTA}] + k_+[AT^B_{e_1e_2B}][AT] - k_-[AT^B_{e_1e_2BTA}] + k_+[ATe_1e_2BTA][B] - k_-[AT^B_{e_1e_2BTA}] + l_+[AT^B_{e_1e_2}][BTA] - l_-[AT^B_{e_1e_2BTA}] + k_+[AT^B_{e_1e_2BT}][A] - k_-[AT^B_{e_1e_2BTA}]$$
